# Smart nanomedicine for lung cancer: Overcoming drug resistance and therapeutic barriers through targeted delivery, phototherapy, and theranostics

**DOI:** 10.1016/j.ijpx.2026.100701

**Published:** 2026-11-03

**Authors:** Viola Colaco, Ashutosh Gupta, Sandesh Ramchandra Jadhav, Aditya Dev Rajora, Moumita Saha, Sangeeta Adile, Amrita Arup Roy, Soji Soman, Sudheer Moorkoth, Deepanjan Datta, Srinivas Mutalik, Bhupendra G. Prajapati, Ritu Kudarha, Namdev Dhas

**Affiliations:** a Department of Pharmaceutics, Manipal College of Pharmaceutical Sciences, Manipal Academy of Higher Education, Manipal, India; b Department of Pharmaceutical Quality Assurance, Manipal College of Pharmaceutical Sciences, Manipal Academy of Higher Education, Manipal, India; c Department of Zoology, Guru Ghasidas Vishwavidyalaya, Bilaspur, Chhattisgarh, India; d Department of Pharmaceutics, SRM College of Pharmacy, Faculty of Medicine and Health Sciences, SRM Institute of Science and Technology, Kattankulathur, Chengalpattu, Tamil Nadu, India; e Shree S. K. Patel College of Pharmaceutical Education and Research, Ganpat University, Kherva, Gujarat, 384012, India; f Faculty of Pharmacy, Silpakorn University, Nakhon Pathom 73000, Thailand; g Centre for Research Impact & Outcome, Chitkara College of Pharmacy, Chitkara University, Rajpura 140401, Punjab, India; h Department of Biotherapeutics Research, Manipal Academy of Higher Education, Manipal, India

**Keywords:** Lung cancer, Nanomedicine, Advanced drug delivery, Cancer therapeutics, Theranostic nanomedicine

## Abstract

Lung cancer is a leading cause of death in the world and is highly heterogeneous with extensive molecular changes and often resistant to therapy. Despite significant advances in clinical management through surgery, chemotherapy, radiotherapy, and immunotherapy, some of these treatments are limited in effectiveness by late diagnosis, systemic toxicity, poor accumulation, molecular heterogeneity, and acquired resistance. This review provides a comprehensive overview of current and emerging therapeutic strategies for lung cancer, with particular emphasis on advanced drug delivery systems and nanomedicine-based approaches. Recent advances in lipid-based nanoparticles, polymeric nanoparticles and micelles, inorganic nanoparticles, and nanocrystals are highlighted for their ability to improve drug solubility, pharmacokinetics, tumor accumulation, cellular uptake, and controlled drug release. Active targeting and stimuli-responsive nanocarriers can improve tumor selectivity, intracellular drug delivery, and drug retention, potentially addressing selected resistance-associated mechanisms. Gene-delivery and genome-editing approaches offer additional opportunities to modulate resistance-related pathways, while photodynamic and photothermal therapies provide complementary strategies for localized tumor treatment. However, the extent to which these approaches reverse established drug resistance varies with the targeted mechanism and the experimental model employed. Distinguishing functional resistance reversal from improved drug delivery and general anticancer activity is therefore essential for assessing their translational potential.

## Graphical abstract

**Figure:**
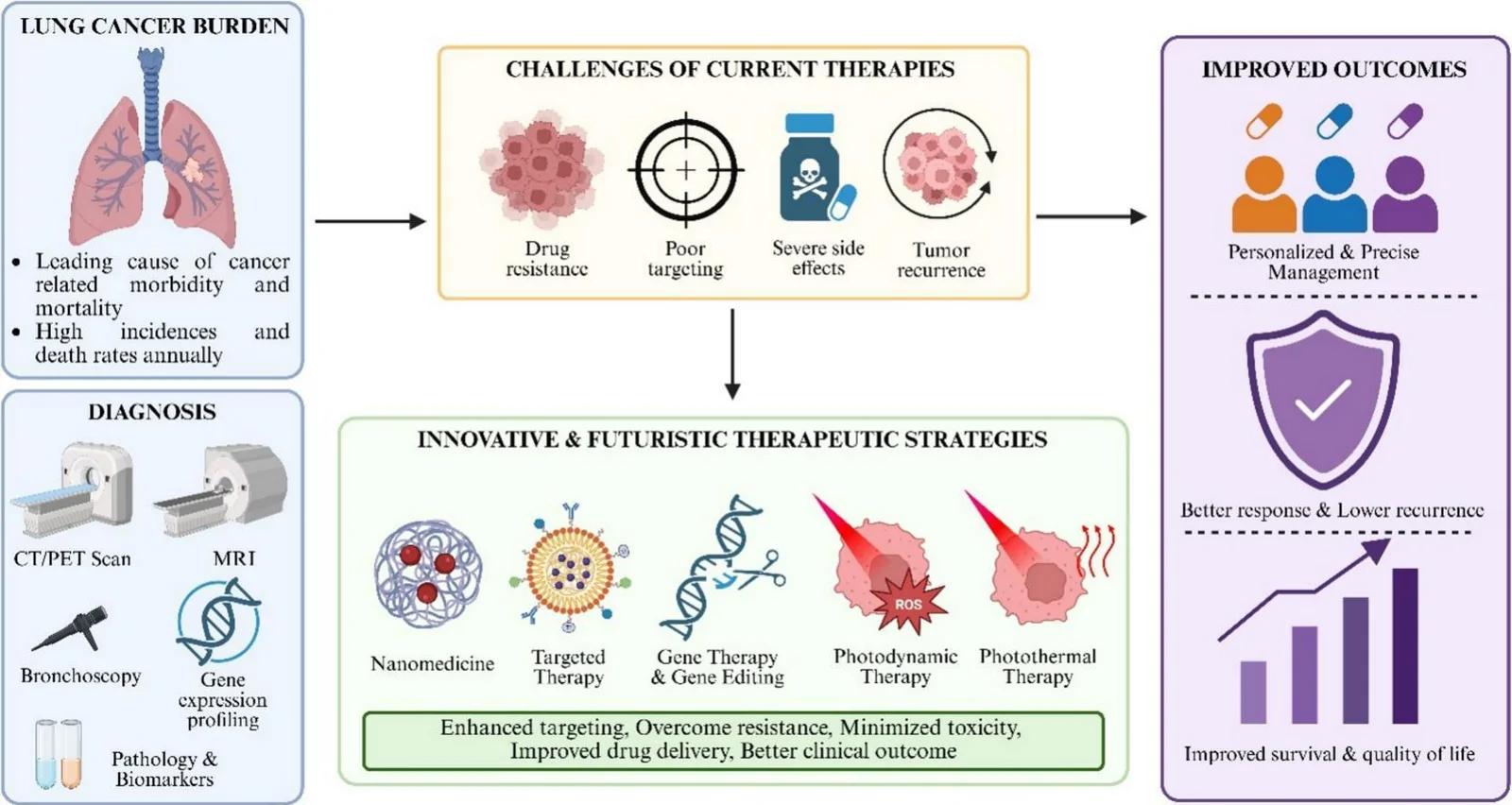

## 1 Introduction

Lung cancer, a malignancy originating in the cells of the lung tissue or bronchi, the passage through which air moves into and out of the lungs. DNA damage of the cells in the airways causes them to multiply rapidly, causing a tumor to grow. It is a major health problem internationally and among the relatively common cancer-related to new cases and deaths in men and women globally. It is estimated to cause 2.21 million cases annually, accounting for 11.4% of all cancer cases, and 1.79 million deaths every year (Chhikara and Parang, 2023). In the United States alone, the American Cancer Society projects 229,410 new cases of lung and bronchus cancer, with an anticipated 124,990 deaths, demonstrating a profound impact on both males and females (Colaco et al., 2024; Siegel et al., 2026). Notably, lung cancer is the second reason of cancer-related mortalities among females after breast cancer (Chhikara and Parang, 2023).

The causes of lung tumors are complex, but exposure to toxic chemicals, especially cigarette smoke, is associated with changes in the cells lining the lungs (dysplasia). The primary causes of lung cancer are genetic factors, smoking habits, environmental factors, pollutants and toxins such as asbestos, arsenic, radiation exposure and organic chemicals, and urbanization (Sharma et al., 2023). The most common risk factor is smoking, which is responsible for about 90% of lung cancer. On the other hand, passive smoking increases the risk of lung cancer by 20–30% (Siddiqui et al., 2024). Lung cancer manifests in distinct histological types. Primarily, it is categorized as small cell lung cancer (SCLC) and non-small cell lung cancer (NSCLC), and carcinoid tumor is a rare variant of lung cancer (M, 2016). NSCLC is the most frequent type of lung cancer (about 85%). Mutations in EGFR and KRAS and expression changes of p16 are common genetic changes observed in NSCLC. However, changes in the genes MYC, BCL2 and TP53 are more commonly linked to SCLC. These changes are often due to long-term exposure to risk factors (Colaco et al., 2024; Sharma et al., 2023; Siddiqui et al., 2024).

The stages of lung cancer define the extent of disease progression, guiding therapeutic approaches and prognostic assessments. Stage 0, also known as in-situ cancer, is restricted to the upper lining of the lungs or bronchus, responsible for localized lesions. Stage I signifies tumors restricted within the lung, while Stage II denotes larger tumors or involvement of nearby lymph nodes. In stage III, the tumor spreads further to the distant lymph nodes, while in Stage IV the tumor metastases to other organs, such as the contralateral lung or distant sites, delineating an advanced and often incurable disease state (Ja et al., 2013). Fig. 1 depicts the survival rate of patients having lung tumor with respect to stages.

**Fig. 1:**
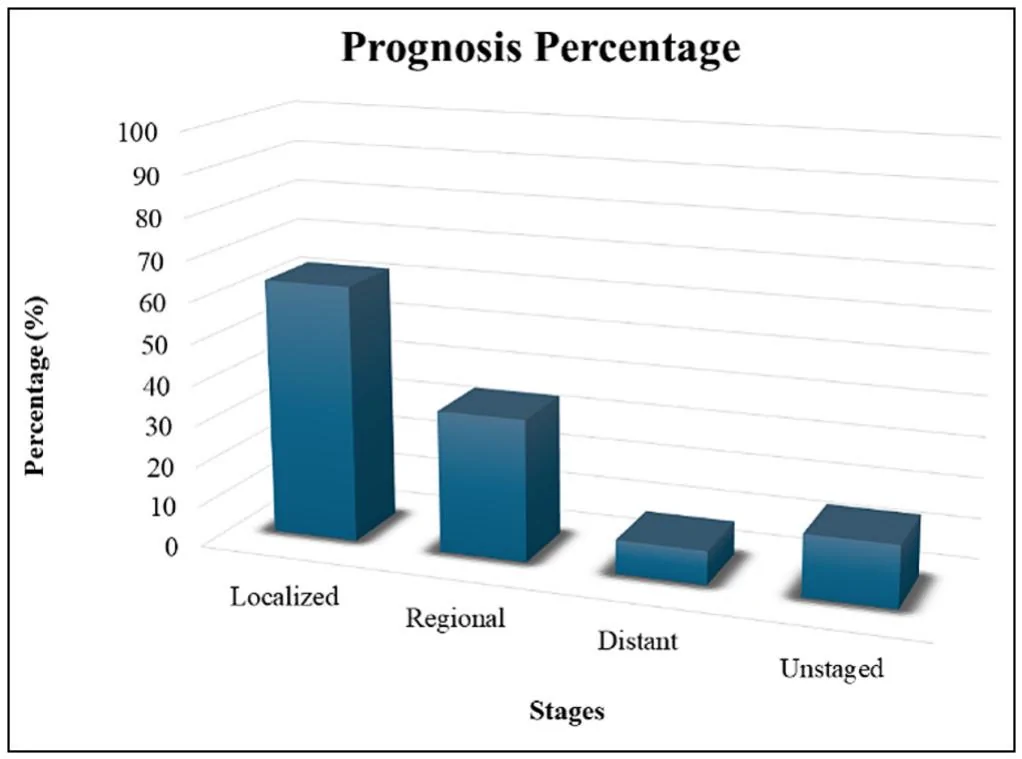
Five-year relative survival by stage at diagnosis for lung and bronchus cancer among patients diagnosed during 2016–2022 in the US SEER 21 registries (excluding Illinois), including all races and both sexes (National Cancer Institute Surveillance, Epidemiology, and End Results Program, 2026).

Existing reviews have discussed nanocarrier platforms, targeted delivery, phototherapy, theranostics, and therapeutic resistance in lung cancer. However, further critical synthesis is needed to connect nanoplatform design with specific delivery barriers and resistance mechanisms, while assessing the strength of experimental evidence and clinical applicability. This review therefore examines nanomedicine-based strategies through a framework linking therapeutic barriers, formulation design, and experimental outcomes. Particular emphasis is placed on distinguishing improved drug delivery and antitumor activity from experimentally demonstrated reversal of drug resistance, alongside evaluating model limitations and translational challenges. This approach aims to clarify the potential and limitations of targeted delivery, phototherapy, and theranostic nanoplatforms for lung cancer treatment.

### 1.1 Types of lung cancer

Widely, lung tumor is classified into two types: NSCLCs and SCLCs. Among all the lung tumor patients, the majority of the cases (near about 80–85%) were of NSCLC type, originating from the lung's central bronchi epithelial cells, stretching to the terminal alveoli (Clark and Alsubait, 2024; PDQ Adult Treatment Editorial Board, 2002). Whereas, 10–15% of the reported cancer incidences were of SCLC type, which spread throughout the body and exhibits extensive necrosis due to its intrusive nature. The histology of SCLC manifests the round to oval-shaped cells with scant cytoplasm. SCLC carcinogenesis involves different pathways that disrupt DNA repair mechanisms, often marked by mutations including lowering of RB1 anti-oncogenes and transformation of TP53 gene. Furthermore, nearly all SCLCs exhibit short-arm omission of chromosome 3p, affecting FHIT anti-oncogenes. These genetic alterations contribute to the diminished pro-apoptotic activity of cancer cells, underscoring the aggressive nature of SCLC (Basumallik and Agarwal, 2024; Raso et al., 2021). Fig. 2 depicts the classification of lung cancer.

**Fig. 2:**
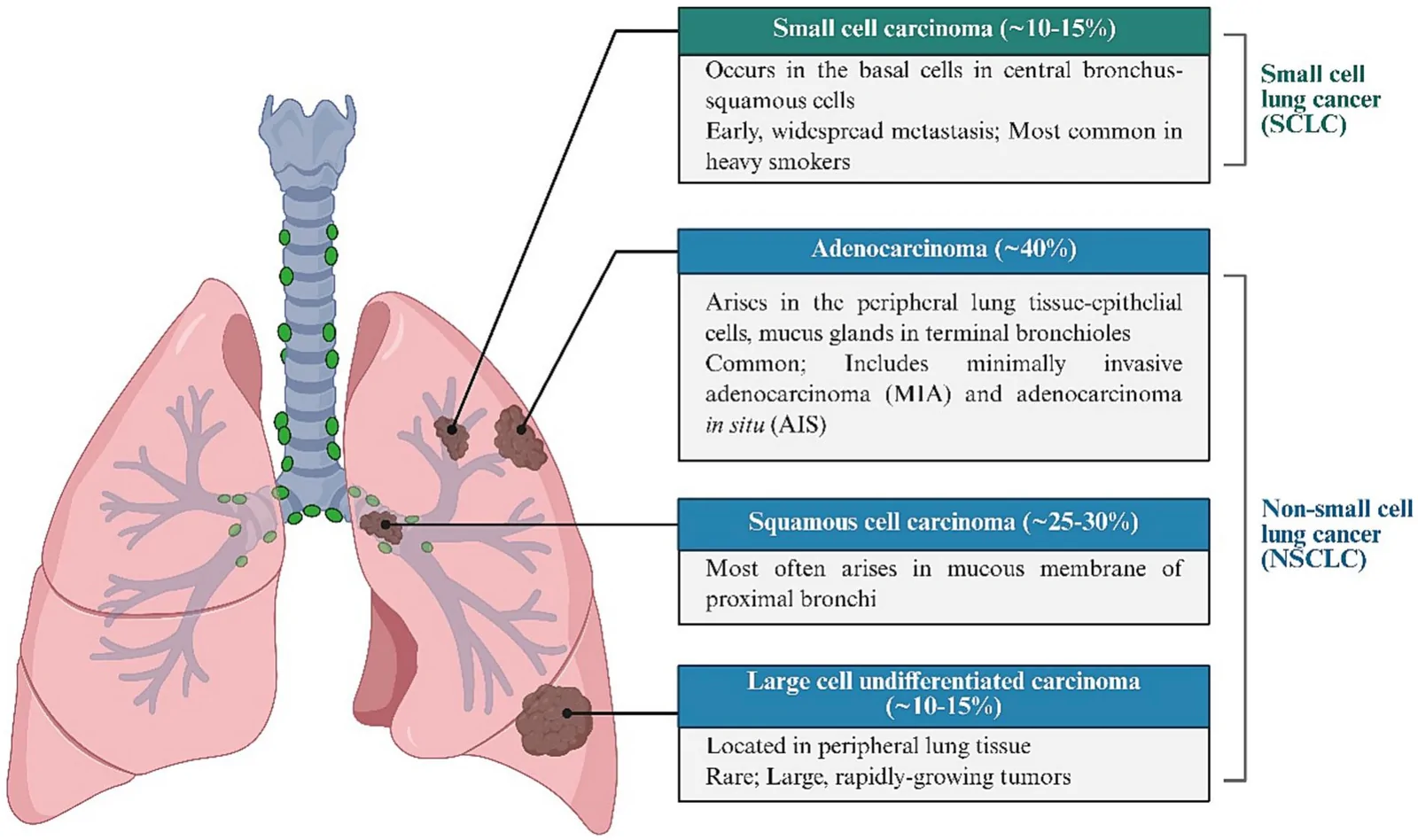
Overview of the major types of lung cancer: SCLC and NSCLC subtypes. created using BioRender.

### 1.2 Epidemiology

#### 1.2.1 Demographic patterns and gender differences

The average age where cases of lung cancer are seen is between 65 and 74 years, with about 35.2% of cases having an average age of 71 years. At the age of 64 years and below, the percentage of new cases is 28.4%, and above 74 years, it's 36.4% (Cancer of the Lung and Bronchus - Cancer Stat Facts, n.d). From the provided data, it is evident that older individuals have an increased chance of lung tumor in comparison to younger individuals. The age-related factors reduce the ability to withstand and repair damaged DNA, decrease metabolite NAD^+^ and shorten telomeres (Siegel et al., 2023; Thandra et al., 2021). Smoking is the leading trigger of lung tumor. Males are at higher risk of diagnosing with lung tumor due to higher tobacco smoking. At the same time, females are at higher risk of adenocarcinoma because it is commonly seen in non-smokers (Thandra et al., 2021). Irrespective of smoking status, women are more susceptible to the certain adenocarcinomas, such as EGFR-positive tumor due to predisposing of CY1A1 and TP53 genes. Hormonal factors, including overexpression of α oestrogen receptors in adenocarcinomas and variables affecting oestrogen exposure like menstrual cycle length and parity, have been implicated, although the association with hormonal replacement therapy and lung cancer risk appears inconclusive based on a meta-analysis of cohort studies (Bae and Kim, 2015; Planchard et al., 2009; Subramanian and Govindan, 2007; Thandra et al., 2021).

#### 1.2.2 Smoking & passive smoke

Cigarette smoke is a complex mixture of toxic constituents known to have negative effects on practically all physiological systems in both active and passive tobacco smokers (Al-Sayed and Ibrahim, 2014). The individuals residing in the regions with high cigarette consumption rates are more prone to lung tumor as smouldering cigarette is the primary cause of lung cancer maturation. Tobacco, major ingredient present in cigarette is reason for the development of tumor in the lungs. In tobacco, nicotine, a biologically active substance, is present in significant amount and is responsible for cigarette addiction. Nicotine binds with the nicotine acetylcholine receptor (nAChR), present in the central nervous system and mimics the action of acetylcholine neurotransmitter and causes variations in gene expression, receptor dynamics, and the level of neurotransmitters. According to USFDA, tobacco smoke contains over 7000 toxic chemicals, among these chemical more than 70 chemical are carcinogenic and can be a root cause of all lung cancer. Long term exposure of these carcinogen causes DNA damage and mutagenesis (Thandra et al., 2021). Passive smoke exposure also demonstrates the dose-dependent correlation with the lung cancer.

Exposure to passive smoke similarly demonstrates a dose-dependent correlation with the risk of lung cancer. Passive smokers may inhale larger quantities of certain carcinogenic chemicals found in passive smoke than active smokers due to the latter's cigarettes having filters. Non-smoking spouses of smokers have been found to be 20–30% more susceptible to lung cancer. Furthermore, exposing pregnant women and children to passive smoking has been associated to the start of chronic health issues (Al-Sayed and Ibrahim, 2014; Simons et al., 2014; Thandra et al., 2021).

#### 1.2.3 Occupational hazards

Exposure to cancerous agents in occupational areas is thought to be a reason for a small fraction of global lung cancer incidence, with workers in the mining industry being more at risk due to welding and asbestos use. Asbestos is a common contributing factor in occupational risks (Spyratos et al., 2013; Thandra et al., 2021). When breathed, asbestos fibres can be a roadblock in the lungs, causing breathing difficulties, coughing, and ultimately pulmonary destruction *Asbestos Exposure and Cancer Risk Fact Sheet - NCI,* 2021).

#### 1.2.4 Air pollution

The quality of breathed air is intricately associated with lung carcinoma. Air pollution due to the combustion by-products from fossil fuels and suspended particulate matter independently is linked with 29% of mortality among all patients of lung cancer. These microparticles gets accumulate in the lungs and cause malignant changes (Bade and Dela Cruz, 2020; Thandra et al., 2021). The residents residing near industrial areas are more prone to developing lung carcinoma due to continuous exposure of carcinogenic substances such as polycyclic hydrocarbons (PAHs) (Thandra et al., 2021).

## 2 Pathophysiology of lung cancer

The occurrence of lung cancer is a multifactorial process and involves the sequential acquisition of genetic and epigenetic changes resulting from chronic exposure to the carcinogen, oxidative stress, chronic inflammation and dysregulated cellular signaling pathways. While cigarette smoking is still the most important etiological risk factor, environmental pollutants, occupational carcinogens including arsenic, asbestos, chromium and nickel, radon exposure and genetic susceptibility also play a significant role in lung carcinogenesis. Damage to DNA, genomic instability and deficits in DNA repair because of these carcinogenic insults lead to malignant transformation of bronchial and alveolar epithelial cells. There are thousands of toxic chemicals in cigarette smoke, including PAHs and tobacco-specific nitrosamines like NNK, which form DNA adducts and cause somatic mutations. These carcinogens also trigger overproduction of reactive oxygen species (ROS), including superoxide (O_2_•^−^) and hydrogen peroxide (H_2_O_2_), which neutralizes the antioxidant capacity within the cell. Oxidative stress is a continuous process that causes damage to DNA, proteins and lipids, interferes with DNA repair system, and triggers mutations leading to the malignant transformation of airway epithelial cells. Carcinogenic effects are also caused by environmental pollutants, occupational toxins and PM that deposits in lung tissues and causes chronic tissue injury (Ashraf-Uz-Zaman et al., 2020; Kunzke et al., 2021).

Persistent oxidative stress and repeated epithelial injury establish a chronic inflammatory milieu that plays a pivotal role in lung cancer initiation and progression. Ongoing exposure to cigarette smoke and environmental toxins results in ongoing recruitment of inflammatory cells such as macrophages, neutrophils and lymphocytes, which in turn lead to sustained production of pro-inflammatory cytokines, e.g. interleukin (IL)-1β, IL-6, IL-17, tumor necrosis factor (TNF)-α and transforming growth factor (TGF)-β. These mediators stimulate proliferation of epithelial cells, inhibit apoptosis, stimulate angiogenesis and promote remodeling of the extracellular matrix. The activation of the NLRP3 inflammasome also enhances the inflammatory signaling by stimulating the production of IL-1β, which has been linked to greater tumor growth, angiogenesis, invasion, and metastases. Moreover, tumor associated macrophages and cancer-associated fibroblasts (CAFs) promote tumor growth by producing growth factors, cytokines, and matrix metalloproteinases (MMPs), and form an immunosuppressive tumor microenvironment (Garon et al., 2020).

Emerging evidence also suggests that alterations in the pulmonary microbiota contribute to lung carcinogenesis. The dysbiosis where bacterial genera like *Streptococcus, Veillonella, Megasphaera*, and *Fusobacterium* are increased results in chronic inflammation by activation of IL-17-producing γδ T cells and MyD88-dependent inflammatory pathways, thus increasing epithelial proliferation and tumor development (Zhao et al., 2021).

The process of normal lung epithelium transforming into invasive carcinoma is a result of a series of genetic and epigenetic changes to oncogenes and tumor suppressor genes. Activating mutations in NSCLC are associated with receptor tyrosine kinase signaling pathways, such as activating mutations in EGFR, HER2, BRAF, MET, RET, ROS1, as well as ALK rearrangements and activating mutations in KRAS. At the same time, the suppression of tumor suppressor genes, including TP53, RB1, LKB1 (STK11), and KEAP1, which are disrupted by loss-of-function mutations, promotes tumor development through the effects on cell cycle, apoptosis, and DNA repair, respectively. These changes trigger several oncogenic signaling pathways such as the PI3K/Akt/mTOR and RAS/RAF/MEK/ERK pathways, which promote the continuous proliferation, metabolic adaptation, resistance to death, increased production of protein, and increased survival of the tumor. Therapeutic resistance and disease progression are partly due to the interplay between these pathways (M et al., 2022; Sanaei et al., 2022; Vathiotis et al., 2021).

The changes in these molecules are accompanied by certain histopathological changes which vary in the different major histological types of lung cancer. SCLC has a unique molecular pathogenesis that involves almost always inactivation of both TP53 and RB1, leading to accelerated growth, genomic instability and early metastasis. Lung carcinogenesis is a process characterised by a series of histological changes, which vary by tumor type. Squamous cell carcinoma typically occurs as a progression of squamous metaplasia, dysplasia, carcinoma in situ, and invasive carcinoma, and usually develops in the central airways of smokers. Adenocarcinoma, in contrast, usually develops in the peripheral parts of the lungs and is a progression of atypical adenomatous hyperplasia, minimally invasive adenocarcinoma and finally, invasive adenocarcinoma. SCLC is derived from pulmonary neuroendocrine cells and has a rapid growth, early lymphovascular invasion (Ashraf-Uz-Zaman et al., 2020; M et al., 2022; Pereira et al., 2021).

As tumors grow, the metabolic requirements of the tumor cause hypoxia in the tumor microenvironment. Hypoxia stabilizes hypoxia-inducible factor-1α (HIF-1α), which in turn promotes the expression of vascular endothelial growth factor (VEGF), platelet-derived growth factor (PDGF), and fibroblast growth factor (FGF) that could lead to abnormal tumor vasculature. In addition, lung cancer cells are also metabolically reprogrammed with heightened aerobic glycolysis (Warburg effect). The ROS and TGF-β pathways drive metabolic shifts in both tumor cells and cancer-associated fibroblasts, which help tumors to quickly proliferate, fibrose and resist treatment (Cabezón-Gutiérrez et al., 2022; Pereira et al., 2021).

Progressing tumors develop mechanisms to escape immune surveillance. Increased expression of programmed death-ligand 1 (PD-L1) suppresses cytotoxic T-cell activity through interaction with PD-1 receptors, enabling tumor immune escape. Other mechanisms of immunosuppression involve the recruitment of regulatory T cells, myeloid-derived suppressor cells, and tumor-associated macrophages, as well as the secretion of anti-inflammatory cytokines that dampen effective antitumor immunity (Jiang et al., 2019; Lin et al., 2024).

As the tumor progresses, epithelial cells become polarized and lose cell-cell adhesion, yet gain the ability to migrate and invade other tissues and organs (epithelial-mesenchymal transition, EMT). The expression of mesenchymal markers like vimentin is promoted by EMT and is increased by TGF-β signaling, inflammatory cytokines and intercellular communication via exosomes. The degradation of the extracellular matrix by the matrix metalloproteinases allows local invasion, intravasation into blood vessels, and dissemination of the tumor cells to distant organs such as the brain, bone, liver, and adrenal glands (Khalili-Tanha et al., 2025).

Chronic lung diseases such as emphysema, chronic obstructive pulmonary disease (COPD), and fibrotic interstitial pneumonia (IIP) increase the susceptibility to develop lung cancer by causing chronic inflammation and tissue remodeling in the lungs. Chronic obstructive pulmonary disease (COPD) significantly increases the risk of lung cancer by persistent oxidative stress, airway remodeling, and chronic inflammatory signaling. Likewise, idiopathic pulmonary fibrosis (IPF) shares several mechanisms of pathogenesis with lung cancer, such as epithelial injury, dysregulated wound healing, TGF-β activation, telomere shortening, and progressive fibrosis, which all lead to the development of cancer. There is also some emerging evidence linking oncogenic viral and bacterial infections, such as human papillomavirus (HPV) and *Chlamydia pneumoniae*, but their role in the pathogenesis of lung cancer is still being investigated. (Pereira et al., 2021; Premachandra and Jayaweera, 2022; Qubo et al., 2023; Tsyganov et al., 2019; Uliński et al., 2022).

## 3 Challenges associated with current lung cancer treatments

Treatment of lung cancer is currently complex, individualized and multidisciplinary and depends on the histological type, the stage of the disease, molecular characteristics of the tumor and patient-specific factors like their general condition and pre-existing comorbidities. The main objective is to improve patients' survival and quality of life by choosing appropriate treatment. Surgery, radiotherapy, chemotherapy, immunotherapy, combination therapy, and supportive care are all key treatments (Park et al., 2019; Valentini et al., 2020).

Surgical therapy is essential for early-stage NSCLC, with a possibility of curative surgery. The surgical method used depends on the size, location and fitness of the patient for the tumor. For operable early stage NSCLC, the common surgical procedure used is lobectomy, which is the removal of one lobe, while pneumonectomy (removal of an entire lung) is the surgery for more advanced disease. Segmentectomy or wedge resection might be considered for patients with limited lung function or smaller tumors. Lymph node dissection may also be performed in conjunction with surgery to assess the extent of disease to determine the need for further treatment (Donington et al., 2022; Howington et al., 2025; Ohtaki et al., 2026). Surgery is usually not recommended in SCLC, however, because of the aggressive and widespread nature of the disease.

In all lung cancer types, radiotherapy plays a vital role as both curative and palliative treatment. Definitive radiotherapy (including stereotactic body radiotherapy (SBRT)) offers medical alternatives to those who are medically unfit for surgery or who refuse surgery and delivers high dose and precise delivery to small, localized tumors in some patients to achieve local control rates comparable to surgery (Iovoli et al., 2023; Jánváry et al., 2025). Radiotherapy is also used as an adjunctive treatment following surgical resection to reduce the risk of local recurrence, especially when the surgical resection has positive margins or there is nodal involvement. Radiotherapy is used palliatively in situations of locally advanced or metastatic disease for symptom control, such as pain, hemoptysis or airway obstruction. Intensity-modulated radiotherapy (IMRT) and proton therapy are new radiotherapy techniques that aim at optimizing tumor targeting while minimizing damage to surrounding healthy tissue (Janopaul-Naylor et al., 2023; Ming et al., 2024; Zerfu et al., 2025).

Chemotherapy is a mainstay of treatment, particularly in advanced NSCLC and SCLC. It includes the systemic application of cytotoxic drugs that are designed to kill fast-growing cancer cells (Bareschino et al., 2011; Kuribayashi et al., 2016). Platinum doublet treatment that combines cisplatin or carboplatin with other drugs, such as paclitaxel (PTX), gemcitabine or pemetrexed, is the first-line treatment for advanced NSCLC. Chemotherapy is also used in the neoadjuvant (before surgery) or adjuvant (after surgery) setting to reduce the burden of disease/inoculum and to eliminate micrometastatic disease, which further improves survival (Horita et al., 2017; Kuribayashi et al., 2016). The main treatment for SCLC is chemotherapy because the disease is aggressive and spreads early. Thoracic radiotherapy has been shown to increase the benefits of chemotherapy in limited-stage SCLC. Although chemotherapy is effective, it can have serious side effects that can affect quality of life and adherence to treatment such as neuropathy, nausea, myelosuppression, and fatigue (Epstein et al., 2022; Sun et al., 2018).

The identification of oncogenic driver mutations in NSCLC has led to the development of targeted therapies to treat the disease. Molecular profiling can help identify actionable mutations, such as those in the epidermal growth factor receptor (EGFR), rearranged anaplastic lymphoma kinase (ALK), and ROS1 fusions (Bui et al., 2018; Li et al., 2013). Targeted drugs are drugs that target these molecular targets and have been shown to have better response rates, longer progression-free survival, and better tolerability than traditional chemotherapy drugs, such as tyrosine kinase inhibitors (TKIs) like erlotinib, gefitinib, osimertinib (for EGFR), and crizotinib, alectinib (for ALK) (Ou et al., 2016; Runzer-Colmenares et al., 2025). Despite this, the emergence of secondary mutations or the activation of alternative pathways is a significant obstacle in clinical practice and the main driver of ongoing efforts to develop new inhibitors and combination therapies.

Immune checkpoint inhibitors (ICIs) that target programmed death-1 (PD-1), programmed death-ligand 1 (PD-L1), and cytotoxic T-lymphocyte-associated antigen 4 (CTLA-4) have reshaped the treatment landscape for advanced NSCLC (Jain et al., 2018; Reck et al., 2022). The immune system continues to respond to tumors, and inhibitory signals are blocking it. Drugs such as pembrolizumab, nivolumab and atezolizumab restore anti-tumor immunity by blocking inhibitory signals that are dampening T-cell activity. Immunotherapy can be utilized alone in those tumors that have high expression of PD-L1 or used in conjunction with chemotherapy to enhance the effect in a larger cohort of patients (Jain et al., 2018; Valecha et al., 2017). While some patients respond to immunotherapy and achieve high overall survival rates, response rates vary, and ongoing research into measuring tumor mutational burden has not shown it to predict response to immunotherapy. IRAEs are less frequently seen than the toxicities of chemotherapy, and are important to monitor and manage (Krieger et al., 2020; Niu et al., 2021).

Combination therapy comprising chemotherapy, targeted therapy, and immunotherapy is becoming increasingly adopted to overcome tumor heterogeneity and resistance mechanisms. For example, the combination of chemotherapy and immunotherapy has proven to be a synergistic approach for the first-line therapy of many patients with advanced NSCLC without actionable mutations. Moreover, the clinical evaluation of the combination of targeted agents with other therapeutic approaches, such as immunotherapy and anti-angiogenic agents, is now underway to maximize therapeutic efficacy and resistance, respectively (Jain et al., 2018; Moya-Horno et al., 2018; Reck et al., 2022).

Supportive care plays an important role in the treatment process for symptom management, to minimize the toxic effects of treatment, and to ensure patient quality of life. This involves pain control, nutrition, respiratory care, psychological therapy and treatment of other conditions. For advanced disease stages, palliative treatments like thoracentesis of malignant pleural effusions, or airway stenting, may be required to symptomatically relieve disease. Despite the advances made in recent years, there remain many hurdles in the treatment arena today, including late diagnosis, tumor heterogeneity, resistance to targeted and immune therapies, therapy-related toxicities, and access to new agents. Current efforts are directed at earlier detection, finding new cancer biomarkers to stratify patients, creating next generation therapies and optimizing combination therapy to enhance the lung cancer patient's response (Avancini et al., 2022; Tiep et al., 2015).

## 4 Nanomedicine-based therapeutic strategies for lung cancer: addressing drug resistance and therapeutic barriers

Traditional treatments often fail to address these dynamic resistance pathways while causing substantial systemic toxicity. Hence, the need for novel, multi-faceted strategies that can circumvent resistance by targeted, specific delivery, genetic interventions and rational drug combinations. Significant progress in nanomedicine, gene editing, immunotherapy, and theranostic platforms provide opportunities for targeting tumors, overcoming efflux mechanisms, interfering with the TME, and obtaining synergistic effects that slow or stop the development of resistance (Colaco et al., 2024; Gupta et al., 2024; Xiang et al., 2024). Fig. 3 depicts the novel strategies in lung cancer treatment.

**Fig. 3:**
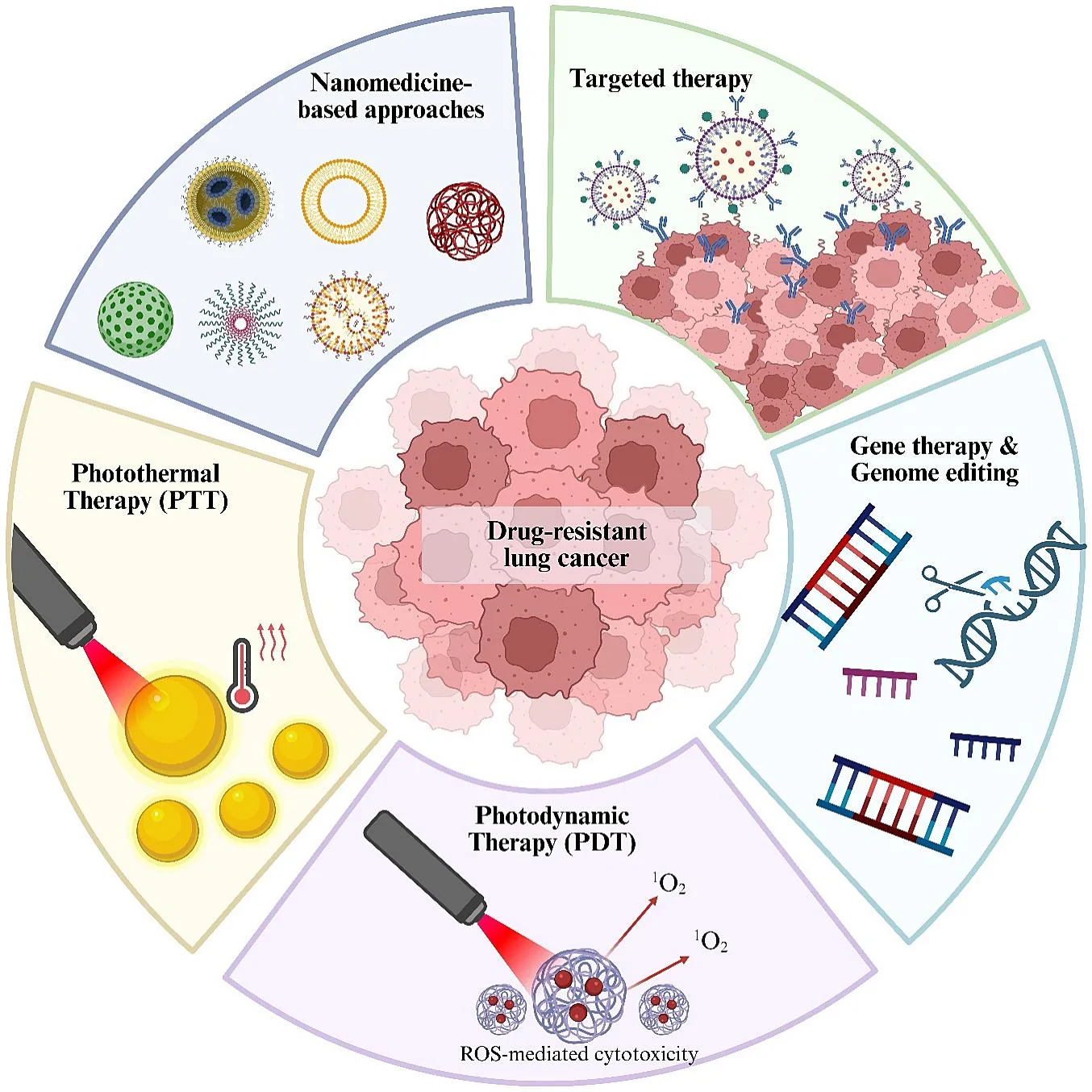
Schematic representation of emerging strategies for overcoming drug resistance in lung cancer. Created using BioRender.

### 4.1 Nanomedicine-based approaches

Nanomedicine-based delivery systems have emerged as an effective strategy for targeted lung cancer therapy by encapsulating anticancer agents within nanocarriers, thereby increasing drug accumulation at the tumor site while reducing systemic toxicity. Compared with conventional free drug administration, nanocarrier-based systems provide improved pharmacokinetic properties, enhanced tumor accumulation through the enhanced permeability and retention (EPR) effect, greater therapeutic efficacy, and lower off-target adverse effects (Allen and Cullis, 2004; Hussain et al., 2007). In addition, these nanocarriers can provide an alternative transport route to deliver drugs to the interior of cells, bypass drug efflux pumps and enable simultaneous delivery of two or more therapeutic agents or nucleic acids (Landesman-Milo and Peer, 2012). A typical nanocarrier comprises a therapeutic payload, a targeting component such as ligands, antibodies, or nucleic acids, and a carrier matrix composed of lipids, polymers, inorganic materials, or dendrimers (Hussain, 2016).

#### 4.1.1 Lipid-based nanoparticles

Liposomes and micelles are the primary lipid-based nanocarriers used in drug delivery. Liposomes are phospholipid bilayer vesicles that can incorporate both hydrophilic (inside the aqueous core) and hydrophobic (inside the phospholipid bilayers) drugs. The ability to tune their physicochemical properties (size, charge, and shape) by changing the phospholipid composition allows them to be made long-circulating, to target tumors, and to be biocompatible (Lee et al., 2023). Numerous chemotherapy drugs, including vincristine and doxorubicin (DOX), have been loaded into PEGylated liposomes and tested in preclinical studies in vitro and in vivo. Because of a passive targeting mechanism that uses EPR, which is driven by the greater vascular permeability characteristic of many solid tumors, PEGylated liposomes can deliver a larger drug load into tumors.

Rahman et al. formulated curcumin (CUR)-loaded solid lipid nanoparticles (SLNs) using glyceryl monostearate as the lipid and Tween 80 as the surfactant, which were selected for their superior drug solubilization and nanoparticle stability (Rahman et al., 2023). The optimized formulation (5% lipid, 2.5% surfactant, and 1% drug) showed spherical morphology which was confirmed by TEM, a particle size of ~115 nm, a PDI of 0.112, and an entrapment efficiency of ~70%. The CUR-SLNs showed an initial burst release with sustained drug release with ~99% cumulative CUR release in 120 h, while only ~10% of the drug was released from free CUR. The formulation exhibited low hemolytic activity and improved CUR stability in PBS. Moreover, CUR-SLNs exhibited significantly higher cytotoxicity against A549 lung cancer cells and cellular uptake (5.2-fold higher than free CUR), indicating effective drug delivery and therapeutic potential. Jarallah et al. investigated GL67/DC-Chol/DOPE liposomal nanocarriers for the delivery of siRNA in A549 lung cancer cells (Jarallah et al., 2023). As the proportion of GL67 increased, particle size also increased, while the zeta potential became more positive and even shifted from negative to positive values at the 3:1 cationic lipid-to-siRNA molar ratio. Higher GL67 formulations (C5 and C6) showed complete encapsulation of siRNA in the gel retardation assays. Evaluation of cytotoxicity indicated high cell viability and good membrane integrity (above 80–85%) for most formulations, thus making them safe. The intracellular delivery of Cy3-labeled siRNA was also enhanced in the final formulation containing 100% GL67 (C6) with ~67% cellular uptake (higher than that obtained with Lipofectamine® (~55%)). Sherif et al. fabricated gefitinib (GEF)-loaded nanostructured lipid carriers (NLCs) for the management of metastatic lung tumors (Sherif et al., 2023). The particle size varied between about 190 to 400 nm, depending on the formulation parameters. In both cases, a significant rise in the SL/LO ratio increased particle size due to increased viscosity of the lipid matrix. The in vitro release data showed a significant difference between the two optimized formulations. GEF-NLCs with the presence of long-chain triglycerides (LCT) showed very fast bursts, and about 70% of the drug was released in the first two hours. However, GEF-NLC also prepared using LCFA showed sustained drug release, where 20% of GEF was released in the first 2 h and 50% in 24 h. The cytotoxicity tests showed that the free GEF and the GEF-loaded NLCs were growth-inhibitory in a concentration- and time-dependent manner in lung cancer cells. The increased cytotoxicity of free GEF could be attributed to the sustained release of the drug from LCFA-based NLCs, as no significant difference was observed at 48 h and 72 h. In another study, a folate-conjugated NLC (FA-DTCR-NLC) co-loaded with docetaxel (DT) and CUR at a synergistic molar ratio (1:2) was successfully developed by Shruti Rawal and colleagues (Rawal et al., 2021). PEGylation and folate functionalization were incorporated to prolong systemic circulation and enable folate receptor-mediated targeting, respectively. Successful folate conjugation was confirmed by FTIR analysis. The optimized FA-DTCR-NLCs exhibited a particle size of ~181 nm, spherical morphology, positive surface charge, high drug entrapment efficiencies (>94%), and sustained, pH-independent drug release for up to 120 h, following Weibull release kinetics. In vitro results showed a significantly higher cytotoxicity, receptor-mediated cellular uptake and intracellular drug delivery in NCI-H460 lung cancer cells than free drugs and NLCs without conjugation on the receptor. Folate receptor mediated endocytosis was confirmed by competitive inhibition with free folic acid. Pharmacokinetic studies indicated that with this formulation there was an extended circulation time, longer half-life, higher systemic exposure, and significantly higher bioavailability than Taxotere®, with the same synergistic DT ratio during circulation. In vivo, FA-DTCR-NLCs significantly decreased tumor volume, amount and incidence, and the histopathological features were improved, and the TNM tumor stage decreased compared with the Taxotere® and unconjugated NLCs.

#### 4.1.2 Polymeric nanoparticles

Polymeric nanoparticles (PNPs) are nanomedical drug delivery systems made of natural or synthetic polymers that are well-established and widely used in polymer-based nanomedicine for improving the efficacy of cancer therapy. A range of polymeric nanocarriers such as polymeric micelles, polymeric nanoparticles, dendrimers, polymersomes, polyplexes, polymer–lipid hybrid systems and polymer–drug/protein conjugates have been extensively studied for targeted cancer therapy. Biodegradable polymers like poly (D, L-lactic acid) (PLA), poly (D, L-lactic-*co*-glycolic acid) (PLGA), poly (ε-caprolactone) (PCL), poly-alkyl-cyanoacrylates and their copolymers, diblocked and multiblocked with poly (ethylene glycol) (PEG). They consist of nanocapsules, dendrimers, colloidal nanoparticles, and polymeric micelles (Chan et al., 2010). Block copolymers composed of two or more polymer chains with varying hydrophobicity can spontaneously self-assemble into polymeric nanoparticles. Moreover, the physicochemical properties of the polymer can be tuned to achieve exact control over the drug release kinetics. Polymeric nanoparticles also have several advantages such as low systemic toxicity, EPR effect, uniform tissue distribution, drug release and long blood half-life in comparison with conventional colloidal systems. All these properties make polymeric nanoparticles a promising platform for the effective treatment of lung cancer (Grewal et al., 2019). Polymeric nanoparticles have also been demonstrated to augment the chemo- and radiosensitizing activity of the anticancer agents. Interestingly, albumin-bound PTX nanoparticle formulation (Abraxane®) has been approved as a first-line regimen with carboplatin for patients with advanced or metastatic NSCLC who are not appropriate for curative surgery or radiotherapy (Y. Liu et al., 2023a; Wang et al., 2010).

A dry powder inhaler (DPI) containing afatinib-loaded PLGA nanoparticles was developed for targeted treatment of NSCLC by Vanza et al., (Vanza et al., 2023). The optimized formulation showed an average particle size of 190–200 nm, drug encapsulation efficiency of about 81% and showed uniform particle size with good drug loading. The nanoparticles were lyophilized using the cryoprotectant trehalose and the anti-adherent L-leucine, yielding a stable dry powder for inhalation. The flowability, drug retention, moisture content, and aerodynamic properties of the dry powder were good; it had an MMAD of ~2.8 μm, an FPF of ~63%, and an emitted dose of >90%, confirming its deep-lung deposition. The formulation was physically stable, exhibited very little aggregation, and demonstrated pH-responsive sustained drug release, with faster release in acidic tumor-mimicking conditions than at physiological pH. The in vitro cytotoxicity studies showed that the nanoparticle formulation exhibited significantly higher anticancer activity than free afatinib with enhanced cellular uptake in A549 lung adenocarcinoma cells. To increase the efficacy of gefitinib therapy and decrease the systemic toxicity, Amin et al. fabricated Gef-CSNPs to deliver the drug to A549 lung cancer cells (Amin et al., 2023). The optimized formulation had an average particle size of ~166 nm, positive zeta potential (+30.2 mV), and high entrapment efficiency (EE ~93%), which confirmed that the formulation was stable in the colloidal state and was efficient in encapsulating the drug. The nanoparticles showed a much higher drug release rate than free gefitinib, according to the diffusion-controlled Korsmeyer-Peppas model. The in vitro cytotoxicity test results showed significantly higher anticancer activity for Gef-CSNPs and significantly lower IC50 value than gefitinib. Flow cytometry was also used to demonstrate the increased ability of the nanoparticles to induce apoptosis and cellular uptake studies showed almost a doubling of gefitinib within the cells after administration of the nanoparticles.

CD56-targeted PLGA-PEG nanoparticles co-loaded with irinotecan (Irt) and Stattic (St) were developed by Arslan et al. for targeted therapy of SCLC (Arslan et al., 2023). The anti-CD56 antibody could be successfully conjugated to the nanoparticles. The optimized formulation exhibited a favorable particle size, a low polydispersity index (<0.3), positive colloidal characteristics prior to conjugation, and a spherical morphology. The results showed controlled release of the drug from the NP vs the free drugs; the best fit model for the release kinetics was the Weibull model. In vitro experiments showed that these targeted co-loaded nanoparticles had a very strong inhibitory effect on the STAT3 signaling pathway, as well as significantly greater cellular uptake mediated by CD56 and cytotoxicity compared to free drugs alone. The biodistribution studies also showed that the nanoparticles with CD56 were preferentially accumulated in the tumor tissue and lungs. The in vivo evaluation revealed better tumor targeting ability, potent anti-tumor effect, low systemic toxicity, and excellent tolerability to non-targeted nanoparticles and conventional drug solution. At the end of therapy, complete tumor eradication was confirmed by histopathological examination, and efficient inhibition of STAT3 pathway in in vivo was shown by western blot.

#### 4.1.3 Polymeric micelles

Polymeric micelles are self-assembled organized nanoscale structure of amphiphilic di/tri block copolymer, above the critical micelle concentration (CMC). They are made up of a hydrophobic core and a hydrophilic shell, which can trap poorly water-soluble drugs and increase their stability and bioavailability (Jin et al., 2022). PEG is the most widely used hydrophilic segment because of its biocompatibility, nonionic nature, and ability to reduce enzymatic degradation through steric stabilization. Other hydrophilic polymers, such as poly(N-isopropylacrylamide) (PNIPAAm), impart thermoresponsive properties, whereas hydrophobic polymers including poly(propylene glycol) (PPG), PCL, PLA, poly(L-histidine) (PHis), and poly(L-aspartic acid) (PAsp) are commonly used to form the micellar core (Alami-Milani et al., 2018; Kim et al., 2005; Wang et al., 2018). Polymeric micelles are promising drug delivery systems for cancer therapy owing to their ability to solubilize hydrophobic anticancer drugs, enhance systemic circulation, improve tumor accumulation, and minimize the adverse effects associated with conventional solubilizing agents (BANDYOPADHYAY et al., 2012; Torchilin et al., 2003).

To combine the delivery of etoposide (ETO) and an alkylated cisplatin prodrug (C6CP) for SCLC therapy, Xiaomeng Wan et al. developed an amphiphilic poly(2-oxazoline) (POx)-based polymeric micelle system (Wan et al., 2018). The optimal co-loaded micelles were found to have a particle size of 75–103 nm with low polydispersity, high colloidal stability and a typical worm-like shape. Co-encapsulation proved to be particularly effective in terms of drug retention (serum binding reduction and slow drug release) compared to single drug micelles. The co-loaded micelles had higher cell uptake, higher cytotoxicity and synergistic anticancer activity in vitro against SCLC and NSCLC cell lines. Good tolerability with decreased renal toxicity compared to free drug combinations was confirmed by toxicological studies. Pharmacokinetic evaluation revealed that both drugs had long plasma half-lives, high tumor accumulation, and high tumor exposure. Co-loaded micelles achieved better tumor growth inhibition, longer survival, higher levels of Pt–DNA adduct formation and apoptosis in multiple lung cancer models than free drugs, single-drug micelles or the mixture of the two, suggesting their potential as a promising nanocarrier for combination lung cancer therapy (Fig. 4).

**Fig. 4:**
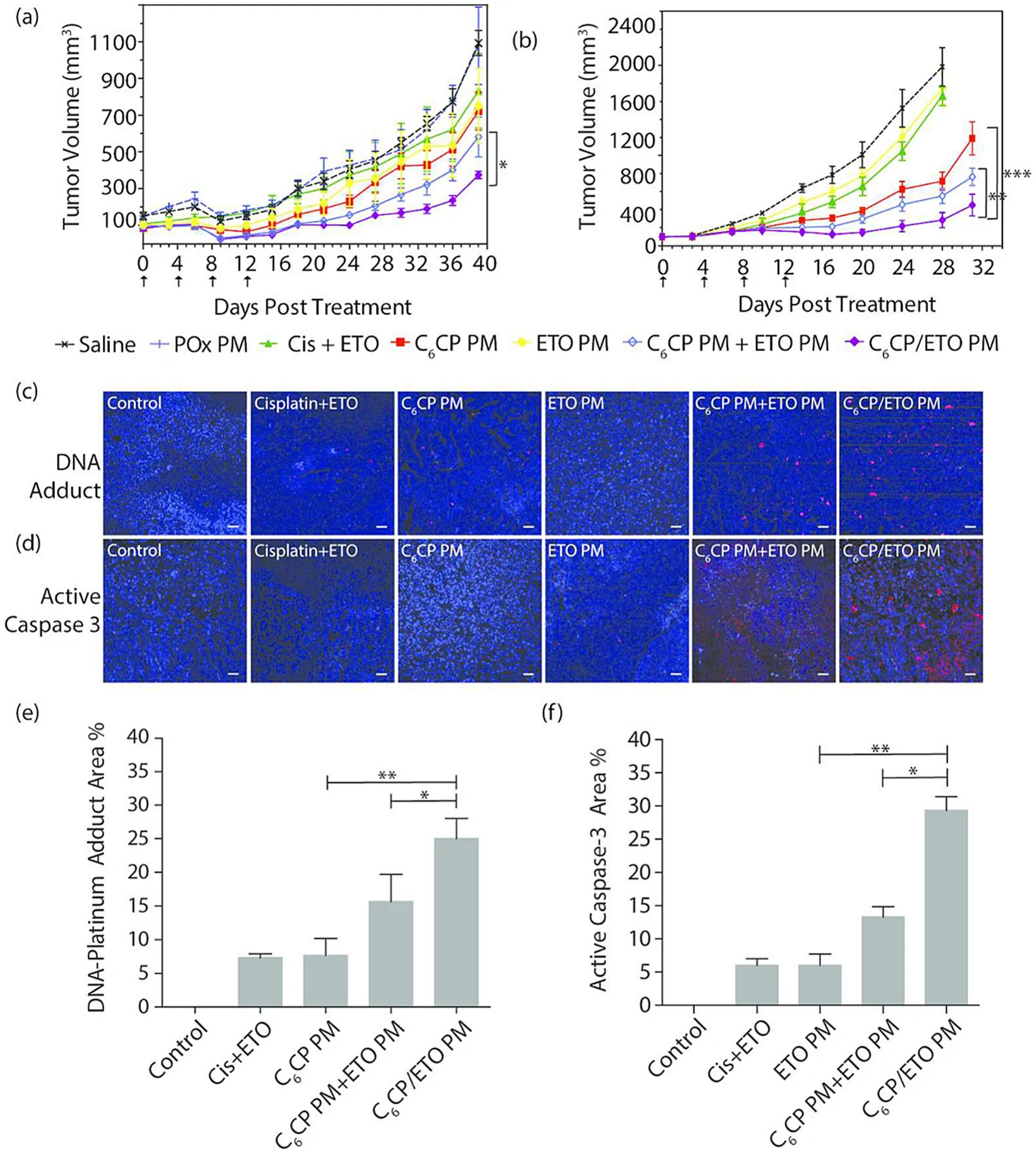
Antitumor effects of the single and co-loaded drug PM in NCSLC and SCLC animal models: (a) A549 and (b-f) H69AR. (a, b) Tumor volume progression, (c, d) histological analysis, and (e, f) quantitation of Pt-DNA adduct (red) and Caspase-3 expression (red) in H69AR tumor sections 2 days after the final treatment. Nuclei were stained with DAPI (blue); scale bar = 20 μm. Data in (a, b) are presented as mean ± SD (*n* = 6). *p < 0.05 versus the C6CP PM + ETO PM combination group in (a) and versus the C6CP PM group and the C6CP PM + ETO PM combination group in (b). * *p* < 0.05; ** *p* < 0.01. For (e, f), five randomly selected microscopic fields were analyzed using ImageJ. *p < 0.05; **p < 0.01. Adapted with permission from (Wan et al., 2018). (For interpretation of the references to color in this figure legend, the reader is referred to the web version of this article.)

An intelligent lung-targeted delivery strategy was developed by Wenxiu He and colleagues using PTX-loaded polymeric micelles in combination with ambroxol (Ax) (He et al., 2020). PTX was encapsulated in amphiphilic PEG-PLA/Pluronic P105 micelles, while Ax was employed to enhance pulmonary surfactant (PS) secretion and inhibit autophagy, thereby improving lung accumulation and sensitizing tumor cells to treatment. The optimized PEG-PLA/P105/PTX micelles exhibited a uniform particle size, a near-spherical morphology, a low critical micelle concentration, and excellent colloidal stability for 48 h. The formulation exhibited sustained PTX release, with complete drug release occurring by 12 h compared with rapid release of free PTX. In vitro, Ax markedly enhanced the cytotoxicity of PTX micelles against A549 lung cancer cells, reducing the IC_50_ by nearly 76-fold at 24 h. In vivo, Ax significantly increased the lung accumulation of PEG-PLA/P105 micelles by enhancing pulmonary surfactant secretion. Pharmacokinetic studies showed prolonged circulation, increased plasma AUC, extended elimination half-life, reduced clearance, and up to fourfold higher PTX accumulation in lung tissue compared with free PTX. Biodistribution studies demonstrated that P105-containing micelles exhibited superior intrinsic lung affinity, with 10% P105 identified as the optimal composition, providing enhanced pulmonary accumulation without compromising micellar stability or drug loading. In an orthotopic A549 lung tumor model, Ax combined with PEG-PLA/P105/PTX micelles produced the strongest tumor inhibition, the longest median survival (52.5 days), and increased LC3 expression, demonstrating that inhibition of autophagy and enhanced pulmonary targeting synergistically improved therapeutic efficacy. In another study, Malik et al., fabricate the TPGS-PLA nano micelles for the targeting the lung cancer (Malik et al., 2024). The findings showed that the formulations had PS between 120 and 190 nm, narrow PDI, and ZP between −18 and − 30 mV. The results of the SEM study showed uniformly distributed, spherically shaped nanomicelles. When PAL-TPGS-PLAM and REG-TPGS-PLAM monotherapies were compared to pure drug dispersions of PAL and REG, the in-vitro drug release profiles of PAL-REG-TPGS-PLAM and these combinations demonstrated a considerably sustained pattern of drug release. When H69PR cells were treated with PAL-TPGS-PLAM, REG-TPGS-PLAM, and PAL-REG-TPGS-PLAM in in vitro cell lines, their cell viability was considerably (*p* < 0.05) lower than that of blank TPGS-PLAM and the corresponding drug dispersion. But compared to the previous groups, PAL-REG-TPGS-PLAM showed more substantial (*p* < 0.01) anticancer outcomes (Malik et al., 2024).

#### 4.1.4 Inorganic nanoparticles

Inorganic nanoparticles (NPs) have emerged as promising platforms for the diagnosis and treatment of lung cancer due to their unique physicochemical properties and versatile surface functionalization. Their surfaces can be readily modified with targeting ligands, while stimuli-responsive drug release and site-specific delivery enhance therapeutic efficacy and minimize systemic toxicity (Jagadish Shetty et al., 2024; Jain et al., 2020). Compared with organic nanocarriers, inorganic nanoparticles offer distinct advantages, including enhanced tumor accumulation and penetration, near-infrared (NIR) optical responsiveness, surface plasmon resonance, efficient radiation-induced secondary electron generation, and facile conjugation with therapeutic and diagnostic agents. Among the various inorganic nanomaterials, gold (AuNPs) and silver nanoparticles (AgNPs) are the most extensively investigated for cancer diagnosis, targeted drug delivery, photothermal therapy, and anticancer treatment owing to their favorable optical, physicochemical, and biological properties (Nahvi et al., 2022).

Polyvinylpyrrolidone (PVP)-stabilized gold nanoparticles conjugated with DOX (DOX@PVP-AuNPs) were developed by Ramalingam et al. to enhance the therapeutic efficacy against A549, H460, and H520 lung cancer cells (Ramalingam et al., 2018).The formulation exhibited pH-responsive drug release, with cumulative DOX release of 91% and 83% at pH 5.0 and 5.8, respectively, compared with only 23% at physiological pH (7.4) after 60 h, indicating preferential drug release under acidic tumor conditions Human lung tumor cell growth was decreased by the in vitro cytotoxic impact of PVP-AuNPs, free DOX, and DOX@PVP-AuNPs at an IC_50_ concentration. Compared with control cells, PVP-AuNPs and free DOX, DOX@PVP-AuNPs can induce both early and late apoptosis in lung cancer cells, increase reactive oxygen species formation, and sensitize the mitochondrial membrane. Furthermore, compared to free DOX and PVP-AuNPs, DOX@PVP-AuNPs significantly upregulate the expression of tumor suppressor genes and cause intrinsic apoptosis in lung cancer cells. Based on the findings, DOX@PVP-AuNPs may be a viable medication delivery method for the successful management of lung tumor in humans. He et al., fabricate the panax ginseng root aqueous extract used silver nanoparticles for the treatment of lung cancer. The monodispersed, spherical nanoparticles, with sizes ranging from 20 to 40 nm, are visible in the TEM picture. Ag NPs' antioxidant potential was evaluated using colorimetric DPPH assays, and their cytotoxicity against the PC14 lung adenocarcinoma cell line and the normal HUVEC cell line was examined in vitro using the MTT technique. An IC_50_ of 157 μg/mL was shown by the Ag NPs nanocomposite against the PC14 cancer cell line. The Ag NPs nanocomposite inhibited the PI3K/AKT/mTOR signaling pathway while inducing LDH release, apoptosis, and ROS levels in PC14 cells (He et al., 2024). Fung et al., fabricate the carbon nanotube for the management of NSCLC. According to the results, MWCNT that was functionalized with OH led to greater and more significant alterations in genes and microRNAs, whereas MWCNT that was functionalized with COOH generated the least amount of biological activity. Analyses of microRNA target filters revealed changed signaling hubs. The only hub that was modified by all three MWCNTs is MYC. The signaling hubs GRB2, AR, TP63, and AGO2 are shared by OH and COOH-functionalized MWCNTs. TP53, STAT3, and BRCA1 are the signaling hubs that were exclusive to OH-functionalized MWCNTs. The in vitro signaling pathways and hubs that we discovered showed a strong correlation with the documented in vivo adverse consequences in mice treated with MWCNTs, including oxidative stress, DNA damage, inflammation, and cancer (Thai et al., 2024).

#### 4.1.5 Nanocrystals

Drug nanocrystals are defined as pure drug particles at the nanoscale, protected from one another by a small quantity of surfactants. Hydrophobic drug delivery is feasible using nanocrystals, which also have optimal drug loading and show promise for industrialization. Improved systemic and oral anticancer activities were demonstrated by drug nanocrystals in laboratory trials (Chen et al., 2017). Miyagi et al. enhanced the chemotherapeutic effect of PTX using the combination therapy with flubendazole nanocrystals (Miyagi et al., 2023). The optimized formulation (15% FBZ, 5% poloxamer 188, 20% L-leucine, and 60% mannitol) demonstrated a three-fold increase in saturation solubility while preserving the crystalline structure of FBZ and exhibiting excellent moisture stability. Cytotoxicity studies in A549 lung cancer cells confirmed that the excipients were non-toxic, whereas FBZ nanocrystals significantly enhanced the anticancer activity of PTX. Pretreatment with FBZ dry powder nanocrystals produced an approximately 1000-fold reduction in the IC_50_ of PTX and demonstrated a synergistic cytotoxic effect, resulting in greater inhibition of tumor cell growth and reduced cell viability during repeated chemotherapy cycles. Xu et al. fabricate LaCoO3 (LCO) lanthanide-based nanocrystals that were rationally engineered as multienzyme-mimicking nanozymes to remodel the tumor microenvironment and induce pyroptotic cell death in lung cancer (Xu et al., 2023). LCO nanocrystals (~285 nm) exhibited uniform morphology, high crystallinity, and homogeneous elemental distribution. They demonstrated multiple enzyme-like activities, including peroxidase (POD), oxidase (OXD), catalase (CAT), and glutathione peroxidase (GPx), enabling enhanced reactive oxygen species (ROS) generation, oxygen production, glutathione (GSH) depletion, and alleviation of tumor hypoxia. In H838 lung cancer cells, LCO nanocrystals showed efficient cellular uptake and negligible toxicity toward normal bronchial epithelial cells, while US activation markedly enhanced ROS production, GSH depletion, mitochondrial dysfunction, apoptosis, and inhibition of clonogenic growth. In vivo, LCO nanocrystals showed high biocompatibility and markedly inhibited tumor cell proliferation (Ki67), with increased TUNEL staining, the activation of NLRP3/Caspase-1 and the substantial reduction in metastatic nodules, especially following US treatment, in both H838 xenograft and metastatic mouse models.

While these nanocarriers have a very high efficiency of drug delivery, passive accumulation is not enough due to tumor heterogeneity and biological barriers. Delivering a drug to the right target cell with the right specificity and efficacy has led to the development of additional active targeting methods.

### 4.2 Critical evaluation of evidence for drug resistance reversal

Chemoresistance in lung cancer develops through several interconnected processes, including ATP-binding cassette (ABC) transporter-driven drug efflux, increased detoxification of therapeutic agents, inhibition of apoptotic signaling, modifications to drug targets, and influences exerted by the tumor microenvironment. Nanomedicine can counteract these mechanisms through approaches such as promoting intracellular drug accumulation, simultaneously delivering cytotoxic drugs and resistance-modulating agents, transporting nucleic acids, directing therapeutics to specific organelles, and enabling drug release in response to biological or external stimuli. Nevertheless, the evidence for genuine reversal of drug resistance depends strongly on the experimental system used and the outcomes measured. Greater tumor accumulation or improved cellular uptake can contribute to enhanced antitumor activity, but such observations do not, by themselves, demonstrate restoration of sensitivity in a pre-existing resistant phenotype (Haider et al., 2022).

Some research provides more direct evidence for the effect of nanocarriers by evaluating the effect of nanomedicines on drug-resistant cancer models. For instance, in one study, DOX and cyclosporin, which inhibits P-glycoprotein (P-gp), were co-delivered via PLGA-based nanoparticles on PTX-resistant A549 cells (A549-TAXOL). The results showed that the delivery system induced higher toxicity compared to DOX and nanoparticle-encapsulated DOX without the inhibitor (Murakami et al., 2011; Xu et al., 2014). Similarly, triphenylphosphine-functionalized PTX liposomes were investigated in drug-resistant A549/cDDP cells. Enhanced cellular uptake, changes in apoptosis-associated proteins, and improved antitumor activity in xenograft models suggest that mitochondrial targeting may help counter resistance-associated survival mechanisms. Nevertheless, the contribution of the carrier, targeting ligand, and therapeutic agent should be considered when interpreting these results (Zhou et al., 2013).

Nanocarrier-mediated gene delivery represents another strategy for addressing resistance pathways that are not solely dependent on drug efflux. A formulation co-delivering survivin small interfering RNA (siRNA) and a cisplatin-derived Pt (IV) prodrug was investigated for platinum-resistant lung cancer. Although the in vitro apoptotic response was limited, the co-loaded formulation produced greater tumor inhibition than free cisplatin in an A549/DDP xenograft model, highlighting the importance of drug-release kinetics, intracellular delivery, and in vivo evaluation (Ma et al., 2021). In another strategy, self-assembled polyjuglanin nanoparticles were employed for the co-delivery of DOX and anti-KRAS siRNA in DOX-resistant A549/DOX and cisplatin-resistant H69/CIS cells. This formulation produced greater cytotoxicity, altered KRAS and P-glycoprotein expression, and inhibited tumor growth, indicating that concurrent targeting of oncogenic signaling and resistance-associated pathways may be a promising approach for overcoming multidrug resistance (Wen et al., 2017). However, the extent to which these molecular changes directly restore drug sensitivity should be evaluated against the functional outcomes reported in each study.

Stimuli-responsive nanomedicines offer additional possibilities, although their effects on resistance must be distinguished from their independent anticancer activity. For instance, hybrid particles constructed from poly(lactic-*co*-glycolic acid)–lipid containing the photosensitizer 5,10,15,20-tetrakis(4-hydroxyphenyl)porphine (pTHPP) showed greater cytotoxic effect in resistant etoposide cells A549RT-eto, illustrating how nanocarriers may improve the activity of poorly soluble therapeutic agents (Pramual et al., 2020). Nevertheless, enhanced cytotoxicity in a resistant cell line does not, by itself, demonstrate reversal of resistance to the original chemotherapy. Such claims require suitable drug-sensitive controls and, where applicable, comparisons with and without the activating stimulus.

### 4.3 Targeted nanocarrier systems

Targeted drug delivery has emerged as a promising strategy to improve intracellular drug delivery in lung cancer, which is relevant to some resistance mechanisms, while improving therapeutic efficacy and reducing systemic toxicity in the treatment of lung cancer. This enables the therapeutic agents to be targeted specifically to the tumor cells, overcoming some of the major drawbacks of conventional therapies, such as the complex anatomy and physiology of the lungs, which make them difficult to penetrate (Gupta et al., 2023; Mansouri et al., 2024; Sharma et al., 2022). Specialized nanoparticles, namely, polymeric nanoparticles, lipid-based carriers, and inorganic nanoparticles, have been designed to pass through the lung cancer microenvironment to deliver therapeutic molecules (Mishra and Singh, 2020). There are 2 ways to achieve targeting: passive targeting, which uses the EPR effect due to leaky tumor blood vessels and dysfunctional lymphatics (BAZAK et al., 2014), and active targeting, which involves surface modification of nanocarriers with targeting ligands or antibodies that bind to receptors that are overexpressed on cancer cells (Choi et al., 2010). In cancer treatment, these systems can be used for direct delivery to tumor sites, avoiding circulation in the body, and result in higher drug concentrations at the tumor site than in normal tissues, thus enhancing the therapeutic index (J. Li et al., 2023b). In particular, targeted delivery can overcome some of the major resistance mechanisms that cause treatment failure, such as multidrug efflux pumps, insufficient intracellular drug concentrations, and off-target effects (Aravindan et al., 2024). Potential targets for ligand-mediated nanoparticle delivery and for active targeting include key receptors, such as EGFR, ALK, ROS1, HER2, PD-L1, and VEGFR (Bethune et al., 2010; Gendarme et al., 2022; Huang, 2018; Iqbal and Iqbal, 2014). Recent advances also incorporate bispecific antibodies and antibody-drug conjugates (ADCs) that simultaneously target multiple resistance pathways.

Miao et al. developed a chitosan oligosaccharide (CSO)-conjugated pluronic P123 to fabricate CSO-modified liposomes (CP20-LSs and CP50-LSs) for targeted delivery of PTX to NSCLC (Miao et al., 2021). The morphology of liposomes was spherical, the average particle size was ~75 nm, and they were highly stable in serum and showed pH responsive drug release with increased drug release under acidic conditions, with a drug loading efficiency of ~10%. Among the formulations, CP50-LSs with higher CSO content showed significantly higher cellular uptake and a 3.2-fold lower IC_50_ in A549 cells than CP20-LSs. CP50-LSs accumulated 2.6- and 1.6-fold more in tumors than unmodified liposomes and CP20-LSs did, respectively, in vivo fluorescence imaging. Mechanistic studies showed that higher CSO content showed the ability to promote greater adsorption of extracellular proteins, especially collagen, which led to greater interaction of liposomes with tumor cells and cellular internalization. In a study by Ibrahim and colleagues, a protocatechuic acid (PCA)-loaded, folic acid-conjugated BSA nanoparticles (PCA-BSA@FA-NPs) were fabricated to target lung cancer (Ibrahim et al., 2025). The nanoparticles showed uniform size, good stability, high drug loading and entrapment efficiency. The nanoparticles exhibited pH-responsive and sustained drug release, with enhanced release under acidic tumor conditions. In vitro, PCA-BSA@FA-NPs showed improved cytotoxicity against A549 lung cancer cells while reducing toxicity toward normal WI38 cells compared to free PCA and DOX. They also demonstrated enhanced antioxidant activity through superior DPPH, SOD-like, and catalase activities. In vivo, oral administration of PCA-BSA@FA-NPs was safe (LD_50_ > 2000 mg/kg), reduced oxidative stress, restored antioxidant enzyme levels, suppressed CD44, NF-κB, FAK, and MAPK expression, decreased tumor burden, and improved lung histopathology, with the high-dose formulation exhibiting the greatest anticancer efficacy. A nintedanib-loaded solid lipid nanoparticles (NIN-SLNPs) and folic acid-conjugated NIN-SLNPs (FA-NIN-SLNPs) were successfully developed by Nalawade et al. with optimized particle sizes of ~198 nm and ~ 220 nm, respectively, and 83% folic acid surface binding (Nalawade et al., 2025). FA-NIN-SLNPs exhibited pH-responsive sustained drug release, enhanced cellular uptake through folate receptor-mediated endocytosis, and superior cytotoxicity and apoptosis induction in A549 lung cancer cells compared to free nintedanib and non-targeted SLNPs. The formulation was hemocompatible and showed improved pharmacokinetic performance, including prolonged circulation time and higher bioavailability (AUC), with preferential lung accumulation in biodistribution studies. Histopathological analysis confirmed reduced systemic toxicity, indicating that FA-NIN-SLNPs are a safe and effective targeted nanocarrier for enhanced NSCLC therapy.

Beyond improving drug delivery, nanotechnology also enables the delivery of nucleic acids and genome-editing systems, thereby expanding its role from targeted drug transport to molecular therapy.

### 4.4 Gene therapy and genome editing approaches

Gene therapy offers a promising molecular approach to overcome drug resistance in lung cancer by directly modifying the genetic drivers of tumor progression and treatment refractoriness. Gene therapy can reverse the oncogenic mutations or restore the function of tumor suppressor genes or even inhibit the expression of resistance-related genes; it may offer long-lasting therapeutic effects without extensive systemic side effects compared with chemotherapy, radiotherapy or surgery. A number of approaches have been tried, such as gene replacement, antisense oligonucleotides, RNA interference (RNAi), and suicide gene therapy (Lara-Guerra and Roth, 2016; Ziółkowska-Suchanek and Rozwadowska, 2025).

Chattopadhyay et al. designed and optimized a chitosan–PLGA nanoparticle (NP) based gene delivery system that co-delivers the tumor suppressor genes miR29A-B1, miR34A and CASP8 for the treatment of NSCLC (Chattopadhyay et al., 2023). By using bioinformatic analysis, it was concluded that CASP8, miR29A-B1 and miR34A were significantly downregulated or genetically modified in NSCLC, leading to impaired apoptosis and poor patient survival. It was found that the fabricated chitosan-PLGA NPs were uniform, biocompatible, and had efficient cellular uptake with pH-responsive DNA release and higher release rates under acidic endosomal conditions (pH 5.0). The NPs showed excellent delivery efficiency of the therapeutic genes into A549 cells and tumor spheroids, resulting in a significant increase in the expression level while also showing low cytotoxicity. Co-delivery of CASP8, miR29A-B1 and miR34A, on the contrary, provided the biggest apoptotic response (51% cell death), significantly more than that obtained with the individual delivery of the genes, and reduced the expression of the anti-apoptotic targets BCL2 and SIRT1.

In another study, a multifunctional protamine-assisted, hyaluronic acid (HA)-modified cationic liposomal vector (PS@HA-Lip) was engineered for targeted delivery of CRISPR/Cas9 plasmid (pMTH1) to disrupt the MTH1 gene in NSCLC, which was developed by Wang and colleagues (Wang et al., 2022). The nanocarrier (~130 nm, PDI ~0.22) exhibited high stability, low cytotoxicity, and efficient plasmid encapsulation. HA modification enhanced CD44 receptor-mediated cellular uptake and tumor accumulation, while protamine promoted nuclear localization of the CRISPR/Cas9 plasmid, resulting in transfection efficiency (~80%) comparable to Lipofectamine 2000. The system achieved efficient MTH1 gene editing (33.1% in vitro; 29.3% in vivo), markedly reduced MTH1 protein expression, inhibited tumor cell proliferation, and induced apoptosis. In orthotopic NSCLC-bearing mice, PS@HA-Lip/pMTH1 selectively accumulated in tumors, significantly suppressed tumor growth (~66.7%), reduced liver metastasis, prolonged survival to 65 days, and showed minimal off-target editing, low immunogenicity, and excellent biosafety (Fig. 5).

**Fig. 5:**
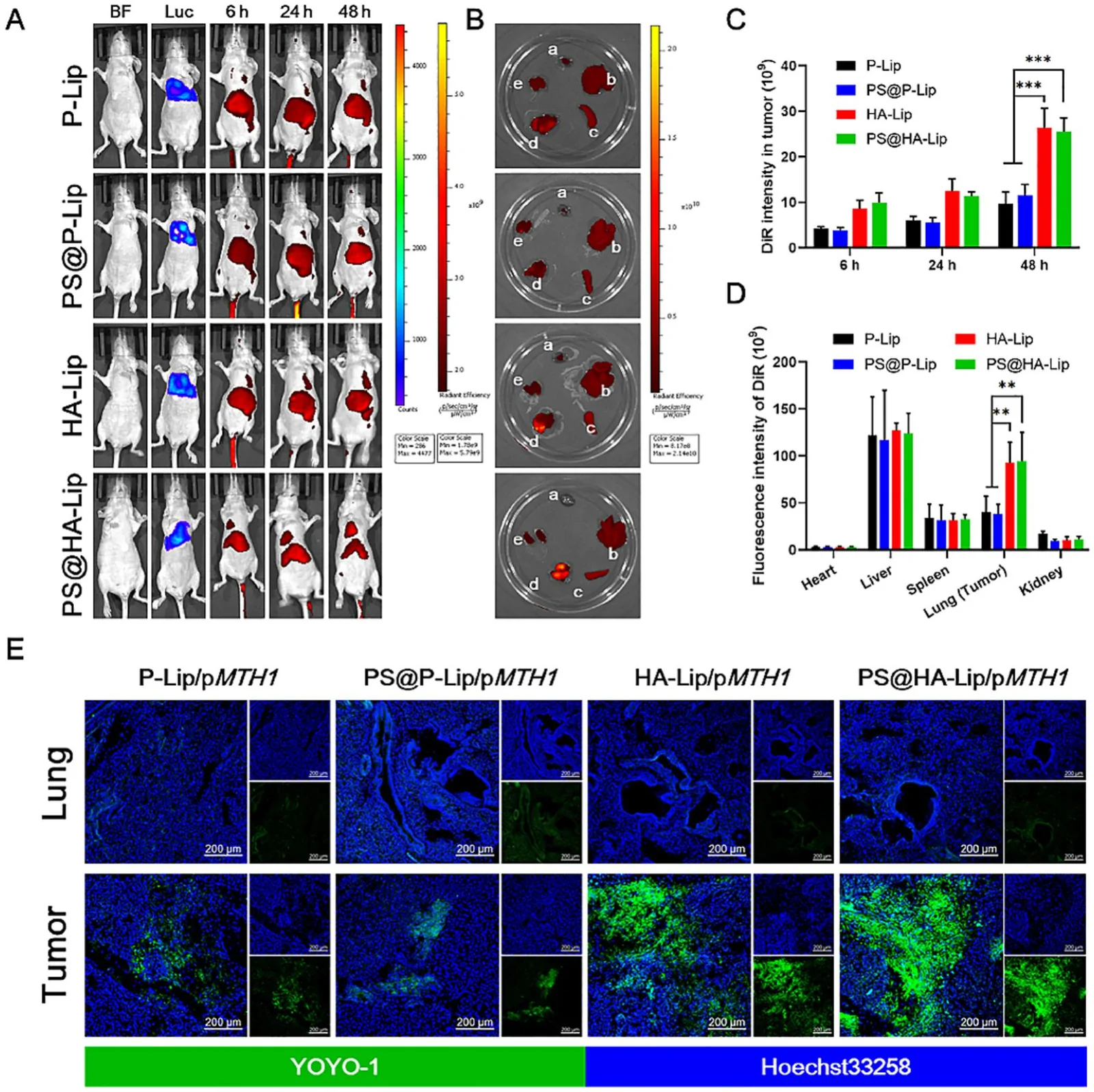
In vivo targeting effect of PS@HA-Lip/pMTH1. (A) Biodistribution of DiR labeled formulations in orthotopic NSCLC-bearing mice at 6, 24 and 48 h post administration. (B) Ex vivo distribution of DiR-labeled formulations in the major organs of NSCLC-bearing mice: heart (a), liver (b), spleen (c), lung/tumor (d), and kidney (e). (C) Quantitative fluorescence intensity of DiR in the lung/tumor at 6, 24, and 48 h following administration of different formulations. (D) Quantitative fluorescence intensity of DiR in the major organs of NSCLC-bearing mice. (E) Biodistribution of YOYO-1 labeled formulations in normal lung and tumor tissue. Data are presented as mean ± SD, *n* = 5, **P < 0.01, ***P < 0.001. Adapted with permission from (Wang et al., 2022).

Li et al. comprehensively evaluated the expression, biological significance, immune relevance, and prognostic value of BUB1 and BUB3 in lung adenocarcinoma (LUAD) using multiple public databases and in vitro validation (X. Li et al., 2023a). BUB1 mRNA was consistently overexpressed in LUAD across TCGA, GTEx, Oncomine, GEPIA, and TIMER datasets, while BUB3 showed elevated expression at both the mRNA and protein levels. Functional enrichment analysis indicated that BUB1/3 are involved in cell cycle regulation, viral carcinogenesis, and AMPK signaling, and their expression correlated with infiltration of B cells, CD4^+^ T cells, CD8^+^ T cells, and neutrophils. Elevated BUB1/3 expression was associated with advanced clinicopathological features, including lymph node metastasis, TP53 mutation, smoking status, and tumor stage. ROC analysis demonstrated strong diagnostic potential, particularly for BUB1, while Kaplan-Meier analysis revealed that high BUB1 and BUB3 expression was associated with poor overall survival, with BUB1 also predicting shorter disease-free survival. In vitro experiments further confirmed the upregulation of BUB1 and BUB3 in LUAD cell lines (X. Li et al., 2023a).

In addition to serving as carriers for therapeutic molecules, nanomaterials themselves can function as therapeutic agents by converting external energy into localized cytotoxic effects.

### 4.5 Photodynamic therapy

A different type of method for treating cancer, called photodynamic therapy (PDT), has been approved for use in clinical trials after it was demonstrated to be successful against a variety of tumor types with minimal adverse events. PDT eliminates tumor cells by using light and non-toxic photosensitizers (PS) inside the tumor and in the blood vessels (Ashkbar et al., 2020; Ostańska et al., 2021). PSs that are activated by light go through energy-transfer cascades that produce reactive singlet oxygen molecules and cytotoxic superoxide anion radicals, which have the potential to kill cells. PDT's anticancer effects are caused by three interrelated processes: direct cytotoxic effects on cancer cells, indirect vascular damage, and the creation of an inflammatory process that may stimulate systemic immunity. The biological action could only be present within the tumor since neovascularization and “leaky capillaries” are characteristics of tumors. This might lead to an increase in photosensitizer concentration in cancerous cells. An increasing number of research supports PDT's capacity to generate strong immunological responses (Kleinovink et al., 2017). A ROS-responsive liposomal nanoplatform (CLL) was developed by encapsulating a luminol-chlorin e6 (Ce6) conjugate, enabling tumor microenvironment-activated photodynamic therapy (PDT) without external light through chemiluminescence resonance energy transfer (CRET) by Yuan et al. (Yuan et al., 2025). CLL generated intracellular ROS and singlet oxygen, resulting in dose- and time-dependent cytotoxicity against A549 lung cancer cells. To enhance therapeutic efficacy, ^177^Lu was incorporated into CLL via DOTA chelation, producing the dual-functional CLL-^177^Lu nanoplatform (~142 nm, >90% radiolabeling efficiency) with excellent colloidal stability. CLL-^177^Lu exhibited enhanced cellular uptake, ROS generation, apoptosis, and synergistic PDT–radiotherapy effects in A549 cells compared with PDT or radiotherapy alone. In lung tumor xenograft and lung metastasis mouse models, CLL-^177^Lu significantly inhibited tumor growth, reduced metastasis, induced apoptosis, suppressed angiogenesis, and promoted M1 macrophage polarization while showing favorable pharmacokinetics, tumor accumulation, and minimal systemic toxicity at therapeutic doses (Fig. 6).

**Fig. 6:**
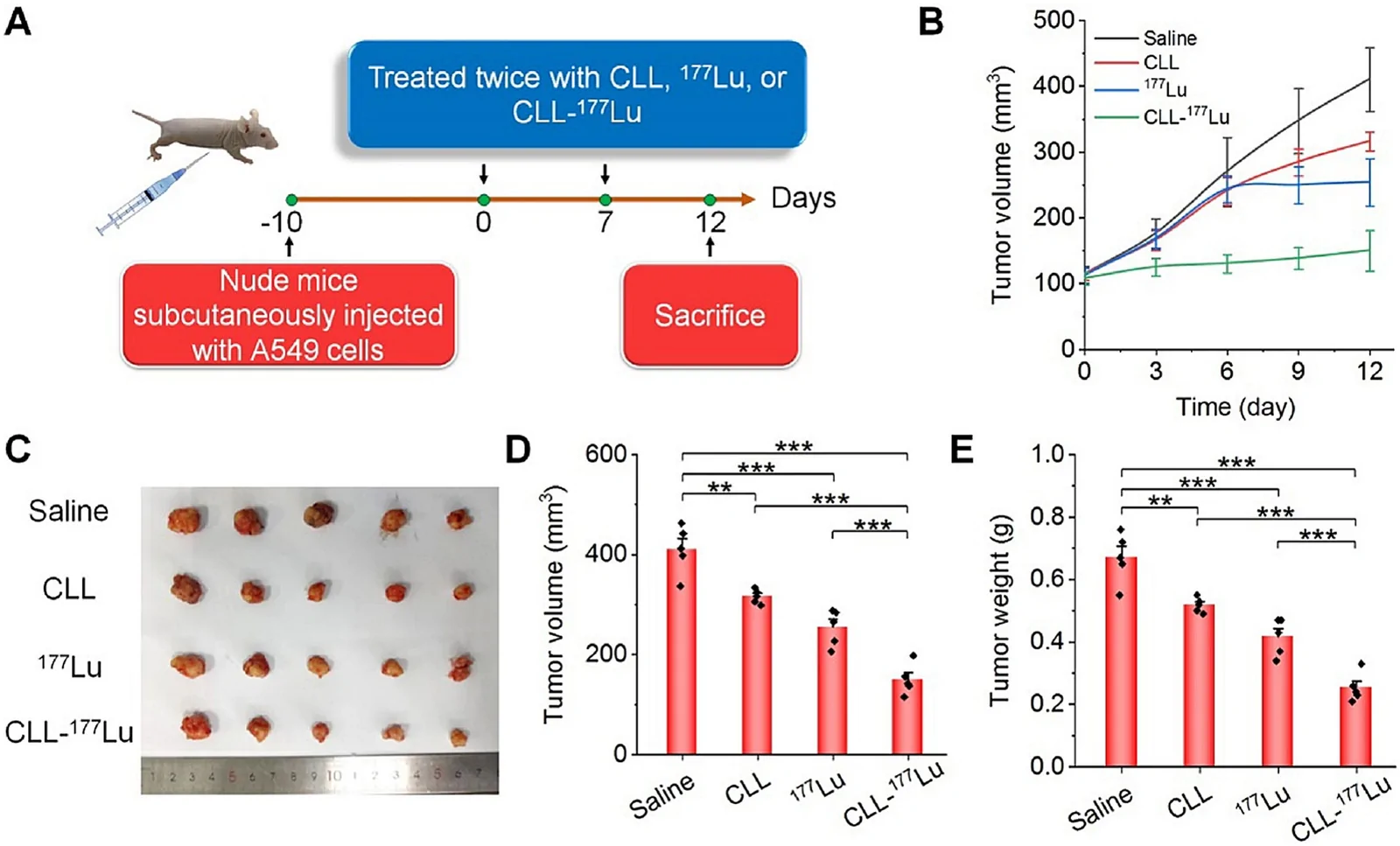
Comparison of antitumor effects of ^177^Lu, CLL, and CLL-^177^Lu in A549 tumor-bearing mice. (A) Schematic representation of the treatment protocol. (B) Tumor growth profiles following treatment with different formulations. (C) Representative images of excised tumors, along with quantitative analysis of (D) tumor volume and (E) tumor weight after the respective treatments. Data are presented as means ± SD (B, D, E, *n* = 5). ∗*p* < 0.05, ∗∗*p* < 0.01, ∗∗∗*p* < 0.001. Adapted with permission from (Yuan et al., 2025).

In another study, two novel porphyrin derivatives, PTA and PTBA, were successfully synthesized and assembled with Zr^4+^ into metal–organic framework nanoparticles, PMOF1 and PMOF2, respectively (Meng et al., 2025). Compared with TCPP-derived PCN224, PMOF1 and PMOF2 exhibited favorable physicochemical properties, including uniform particle size, high colloidal stability, and red-shifted optical characteristics. Under laser irradiation, both nanoparticles generated significantly higher levels of ROS and singlet oxygen, with PMOF1 showing the greatest photodynamic activity. Nanoparticle formulation markedly enhanced cellular uptake and tumor retention compared with free porphyrins. Consequently, PMOF1 and PMOF2 demonstrated superior phototoxicity, inducing greater inhibition of cell viability, colony formation, and increased apoptosis in H520 lung squamous cell carcinoma cells. In vivo, both nanoparticles significantly suppressed tumor growth under laser irradiation without evident systemic toxicity, with PMOF1 achieving the strongest antitumor efficacy. Moreover, PMOF1 and PMOF2 exhibited broad-spectrum photodynamic anticancer activity across multiple cancer cell lines, consistently outperforming PCN224. Bender et al., fabricated CUR-loaded DPPC/cholesterol liposomes for pulmonary photodynamic therapy (Bender et al., 2024). TFH and P-DC liposomes exhibited smaller particle sizes and lower PDI than LF-DC. Nebulization did not significantly alter particle size, zeta potential, or aerosol output, confirming good nebulization stability. Morphological analyses (AFM and TEM) showed spherical liposomes, with TFH producing predominantly unilamellar vesicles, LF-DC less uniform vesicles, and P-DC multilamellar structures. In A549 cells, CUR-loaded liposomes enhanced ROS generation, mitochondrial membrane depolarization, cellular uptake, and phototoxicity compared with free CUR, while exhibiting minimal dark toxicity. TFH and LF-DC formulations demonstrated superior photodynamic efficacy over P-DC, likely due to improved cellular uptake and more favorable lamellar structures. All formulations were biocompatible in the HET-CAM assay, causing no irritation. In the CAM xenograft model, nebulized TFH and LF-DC liposomes significantly reduced tumor metabolic activity and cell proliferation, as confirmed by PET/CT, H&E, and Ki-67 staining.

While PDT relies on reactive oxygen species generation, another complementary light-triggered modality utilizes photothermal conversion to induce localized hyperthermia.

### 4.6 Photothermal therapy

Photothermal therapy (PTT) is a minimally invasive, light-based therapy that is based on the conversion of absorbed light energy, usually in the NIR region, into heat at the site of the tumor to selectively destroy the target cells. Thermal treatment for cancer was derived from hyperthermia, wherein temperatures around 40–45 °C cause irreversible changes in protein structure, disrupt the tissue membranes, and trigger apoptosis in cancerous cells with minimal damage to healthy tissue (Bhole, 2021; Chu et al., 2022). PTT uses biocompatible nanomaterials that have high photothermal conversion efficiency, so that precise spatiotemporal heating is possible when irradiated by an NIR laser. The NIR biological window (700–1350 nm), especially the second NIR (NIR-II, 1000–1350 nm) window, exhibits a better tissue penetration and less light scattering, which makes it possible to treat deeper tumors more effectively. Gold nanoparticles, carbon-based nanomaterials, transition metal sulfides (TMSs), and conductive polymers have been identified as effective photothermal agents because of their high NIR absorption and high photothermal conversion efficiency (Bhole, 2021; Ren et al., 2022).

A multifunctional biotin-functionalized MoS₂ nanoplatform (MoS₂-PEG-Biotin) was developed for the targeted co-delivery of CUR and erlotinib (Er), enabling synergistic chemotherapy combined with NIR-mediated photothermal therapy (PTT) for lung cancer by Chen et al. (Chen et al., 2023). The PEGylated nanosheets exhibited a sheet-like morphology (~120 nm), increased thickness (~2.3 nm), excellent colloidal stability in physiological media, and improved dispersibility compared with unmodified MoS_2_. MoS_2_-PEG-Biotin showed excellent photothermal performance, exhibiting concentration- and laser power-dependent temperature elevation under 808 nm NIR irradiation with high photothermal stability. CUR and Er were successfully co-loaded with loading capacities of 22.3% and 10.1%, respectively. Drug release was minimal under physiological conditions but significantly accelerated upon NIR irradiation, demonstrating laser-responsive controlled release. Biotin-mediated targeting significantly enhanced cellular uptake and intracellular drug accumulation in A549 lung cancer cells. In vitro, the nanoplatform showed superior cytotoxicity and apoptosis, particularly under NIR irradiation, owing to the combined effects of targeted chemotherapy and PTT. Biodistribution studies revealed preferential tumor accumulation of the biotin-modified nanoplatform, with 2.08–4.15-fold higher tumor uptake than non-targeted nanoparticles. In vivo, NIR irradiation generated tumor temperatures above 48 °C, enabling effective photothermal ablation. Consequently, MoS₂-PEG-Biotin-CUR/Er + NIR achieved the greatest antitumor efficacy, with 95.6% tumor growth inhibition, extensive tumor necrosis, and no significant systemic toxicity or organ damage, demonstrating its potential as a safe and effective targeted chemo-photothermal therapeutic platform for lung cancer (Fig. 7).

**Fig. 7:**
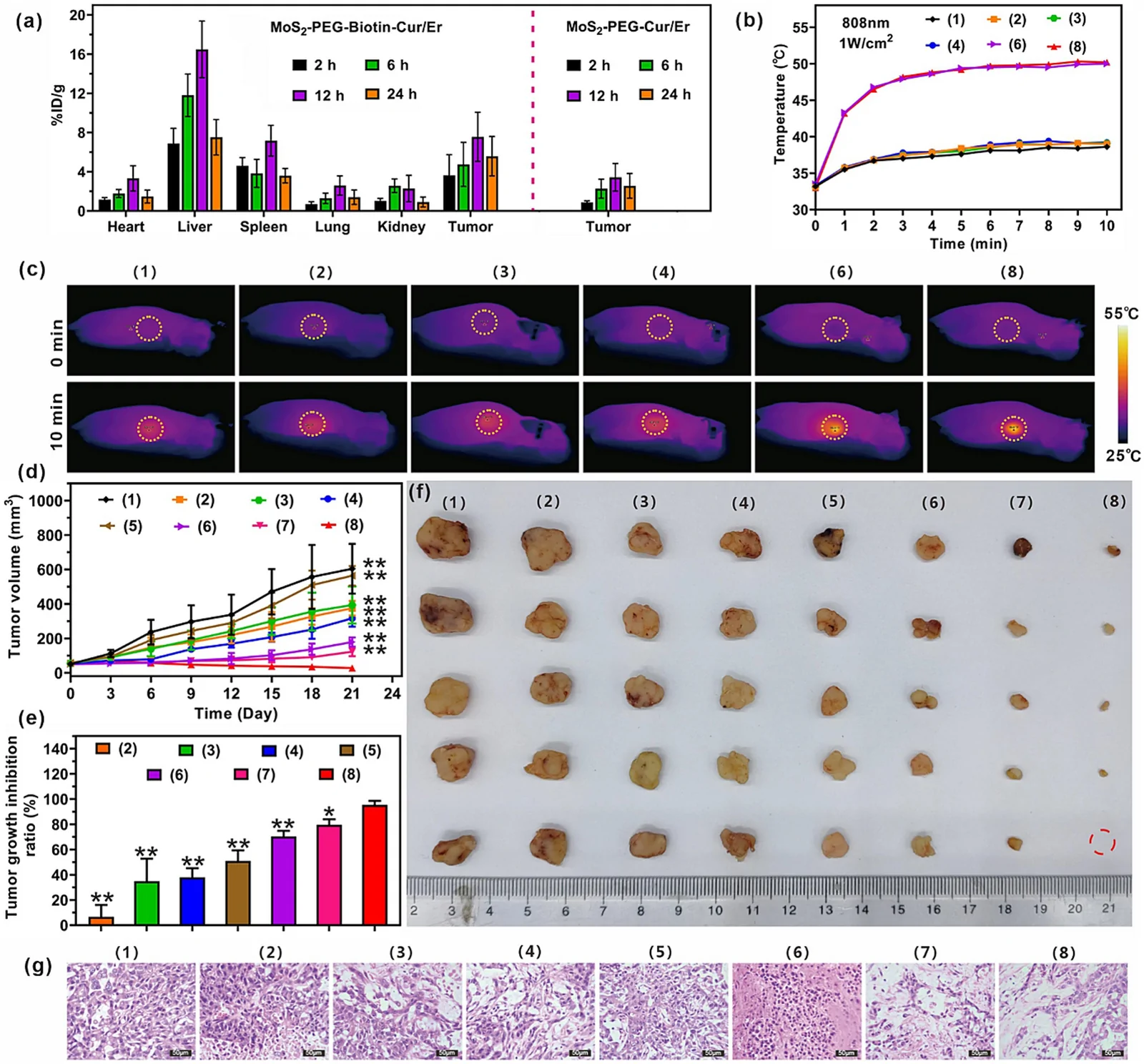
Mice were divided into eight groups: PBS (control), MoS2-PEG-Biotin, CUR, Er, CUR + Er, MoS2-PEG-Biotin + NIR, MoS2-PEG-Biotin-CUR/Er, and MoS2-PEG-Biotin-CUR/Er + NIR, (a) Tissue biodistribution of Mo^2+^ determined by ICP-MS after intravenous administration of MoS₂-PEG-Biotin-CUR/Er and MoS₂-PEG-CUR/Er. (b, c) Tumor temperature changes and corresponding thermal images during NIR irradiation. (d) Tumor growth curves over 21 days. (e) Tumor growth inhibition rates. (f) Representative tumor photographs after treatment. (g) H&*E*-stained tumor sections. Significant differences between the MoS₂-PEG-Biotin-CUR/Er + NIR group and all other groups are indicated as **p < 0.01. Adapted with permission from (Chen et al., 2023).

Maryam Deinavizadeh and colleagues developed gold nanorod-supported thiolated MCM-41 nanocomposite (AuNR@S-MCM-41) as a multifunctional drug delivery platform for DOX, enabling pH/NIR dual-responsive drug release and synergistic chemo-photothermal therapy against lung cancer (Deinavizadeh et al., 2024). AuNRs were successfully immobilized onto thiolated MCM-41 through Au—S bonding, followed by DOX loading via electrostatic interactions. AuNR@S-MCM-41 exhibited excellent photothermal conversion under 808 nm NIR irradiation with concentration-dependent heating. The nanocomposite achieved a DOX loading capacity of 26.36 wt% and entrapment efficiency of 48.27 wt%, while exhibiting pH- and NIR-responsive drug release, with maximal release under acidic conditions and laser irradiation. Cellular uptake studies confirmed concentration-dependent internalization in A549 lung cancer cells. In vitro assays demonstrated excellent biocompatibility of the carrier, whereas DOX-loaded nanocomposites significantly enhanced cytotoxicity. Upon NIR irradiation, AuNR@S-MCM-41-DOX produced superior anticancer activity through synergistic chemotherapy and photothermal therapy, resulting in markedly reduced cell viability compared with either treatment alone.

To combine the synergistic effect of chemotherapy and photothermal therapy (chemo-PTT) for the treatment of lung cancer, multifunctional human serum albumin-based gold nanoshells (HCP@GNSs) were developed for synergistic drug delivery and photothermal therapeutic effects (Yang et al., 2023). HCP@GNSs exhibited strong NIR absorption, concentration- and laser power-dependent photothermal heating, and outstanding photothermal stability under repeated irradiation. The in vitro results showed high biocompatibility, low hemolytic activity, efficient cellular uptake as well as increased cytotoxicity after NIR irradiation due to combined effects of cisplatin chemotherapy and photothermal ablation. In vivo, HCP@GNSs induced efficient hyperthermia under NIR irradiation, leading to efficient tumor regression, 60% tumor clearance, large tumor necrosis area and negligible systemic toxicity. Additionally, the chemo-photothermal treatment resulted in infiltration of the tumor by immune cells such as B cells, dendritic cells, and NKT cells, which suppressed distal tumor growth. The key studies investigating nanoparticle-based targeted therapeutic strategies for lung cancer are summarized in Table 1. Studies were selected to represent diverse nanocarrier platforms and approaches to tumor targeting, overcoming delivery barriers, immune modulation, and combination therapy. Inclusion required evaluation in a lung cancer model and reporting of therapeutic outcomes.

**Table 1:** Nanoparticle-based targeted therapeutic strategies for lung cancer.

Nanoparticle type	Therapeutic Agent	Target/Ligand/ Surface modification	Lung cancer model (Cell line/ Animal model)	Route of administration	Outcome	Ref
Human Serum Albumin Nanoparticles (CTX-HSA-NPs) combined with Lipid Nanoparticles (TGFβ-1 siRNA LNP)	Cabazitaxel (CTX) and TGFβ-1 siRNA	Surface modification with 4 lipids (DSPC, DLin-MC3-DMA, PEG2000-c-DMG, Cholesterol); Target: TGFβ-1 mRNA gene silencing	Cell line: A549, PTX-resistant A549/T Animal model: BALB/c nude mice	Intravenous (Tail vein)	Enhanced antitumor efficacy in PTX-resistant NSCLC, low toxicity, effective gene silencing of TGFβ-1, and lower expression of P-gp avoiding drug resistance.	Tan et al. (2023)
Solid Lipid Nanoparticles (PC-SLNs)	PTX and CUR	1,2-distearoyl-sn-glycero-3-phosphoethanolamine-N-[methoxy (polyethylene glycol)-2000 (DSPE-PEG2000), Hydrogenated soybean phospholipids (HSPC), Polyvinyl pyrrolidone k15 (PVPk15) modification	Cell line: A549 Animal model: Nude BALB/c mice xenograft	Intraperitoneal	Achieved high tumor suppression rate (78.42%), prevented P-glycoprotein efflux, reversed multidrug resistance (MDR), and downregulated the NF-κB pathway.	Pi et al., 2022)
Mannose-modified Selective Organ-Targeting Lipid Nanoparticles (siSPP1-mIFNγ@mLNPs)	SPP1-siRNA and Interferon-γ (IFNγ) mRNA co-loaded	Mannose-modified (DMG-PEG-Mannose) with 40% DOTAP for pulmonary macrophage targeting	Cell line: LLC, LLC-OVA-Luc, TC-1, 4 T1 Animal model: C57BL/6 J and BALB/c mice	Intravenous (Tail vein)	Reprogrammed M2-like SPP1 macrophages to M1-like phenotype, enhanced antigen uptake, boosted anti-tumor immune responses, and significantly inhibited orthotopic tumor growth and lung metastases when combined with anti-PD-1.	Liang et al. (2026)
Chitosan-Zoledronic Acid Nanoparticles (MFCSZA0.5)	Zoledronic acid (ZA)	Chitosan (CS) polymeric matrix via ionic gelation (FRR = 0.5 via microfluidics)	Cell line: A549	In vitro evaluation	Demonstrated significantly greater antitumor activity and apoptosis induction compared to bulk nanoparticles and free ZA; achieved higher drug entrapment efficiency (96%) and gradual, sustained drug release.	Khayati et al. (2025)
mPEG-PLGA Polymeric Nanocarriers (Nanoparticles, Polymersomes, and Micelles)	PTX	MPEG-PLGA diblock copolymers (mPEG_2000_-PLGA_28000_, mPEG_2000_-PLGA_5000_, mPEG_2000_-PLGA_2000_)	Cell line: A549 Animal model: BALB/c nude mice	Intratracheal instillation	Micelles showed the best mucus penetration, tumor spheroid penetration, and lymphatic distribution. Polymersomes provided superior lung retention by overcoming mucus barriers. Rigid nanoparticles showed the strongest cellular uptake. All structures significantly inhibited orthotopic tumor growth in vivo.	Liu et al. (2026)
Cancer Cell Membrane-Camouflaged Nanoparticles (AM@PTX-NPs)	PTX	A549 lung cancer cell membrane cloaking on PLGA core (retaining CD47 and E-cadherin)	Cell line: A549 Animal model: Male BALB/c nude mice	Intravenous (Tail vein)	Displayed homologous tumor-targeting capacity, immune evasion from macrophages, increased cellular uptake, sustained drug release, and a high tumor growth inhibition rate (73.00%) with good safety.	Zhou et al. (2024)
Chitosan-Coated Bacterial Minicells (Minicell@CS-DOX-FA)	DOX	Protein-assisted Chitosan (CS) coating with Folate (FA-PEG-NHS) surface modification	Cell line: LLC Animal model: Female C57BL/6 mice	Intravenous	Enhanced tumor-targeting via folate, induced immunogenic cell death (releasing CRT, HMGB1), activated the cGAS-STING pathway via ROS and mtDNA release, promoted dendritic cell maturation, recruited CD4^+^ and CD8^+^ cytotoxic T cells, and significantly inhibited tumor growth.	Feng et al. (2024)
Superparamagnetic Iron Oxide Nanoparticles in Core-Cross-Linked Polymer Micelles (SPION-CCPMs)	Iron Oxide (Fe_2_O_3_/SPIONs)	Polysarcosine-*block*-poly(S-ethylsulfonyl-L-cysteine) copolymer shell cross-linked with dihydrolipoic acid	Cell line: LLC, Eml4-Alk Animal model: C57BL/6 N and FVB mice (Eml4-Alk driven LUAD)	Intratracheal instillation	Reprogrammed tumor-associated macrophages (TAMs) toward a pro-inflammatory phenotype, induced oxidative stress/DNA damage in cancer cells, recruited CD8^+^ cytotoxic T cells, and halted tumor regrowth post-crizotinib treatment.	Horvat et al. (2024)
Exopolysaccharide-Coated Selenium Nanoparticles (EPS-SeNPs)	Selenium (Se)	Capped/stabilized with bacterial exopolysaccharide (EPS from *Lactiplantibacillus plantarum* strain A2)	Cell line: A549	In vitro evaluation	Demonstrated potent antibacterial/antifungal activity, strong antioxidant effect (97.4%), inhibited A549 cell proliferation via S-phase arrest (IC50 = 5.324 μg/mL), and induced apoptosis by activating Bax/Caspase-3 while downregulating Bcl2.	Shehata et al. (2023)
Metal Nanoparticles (NCPA-capped AuNPs & AgNPs)	Gold (Au) or Silver (Ag)	Capped with N-cholyl D-penicillamine (NCPA)	Cell line: A549	In vitro evaluation	Exhibited low hemotoxicity to human RBCs and potent dose-dependent cytotoxicity against A549 cells (IC50 = 60 μg/mL for AuNPs and 50 μg/mL for AgNPs) via S-phase cell cycle arrest and apoptosis induction.	Elumalai et al. (2023)
Afatinib Gold Nanoconjugates (Afb-AuNPs)	Afatinib analog (Afb-A)	Covalently attached via cyclic disulfide anchor and functionalized with HS-PEG-COOH	Cell line: PC-9, A549	In vitro evaluation	Up to 3.7-fold increase in potency compared to free drug, significantly inhibited cancer cell proliferation, maintained biocompatibility/non-inflammatory profile in normal TT1 cells, and demonstrated acid-responsive drug release.	Cryer et al. (2019)
RGD Receptor-Targeted PLGA Nanoparticles (CPFT-RGD)	Cisplatin (CDDP) and Upconversion Nanoparticles (UCNP) co-loaded	Pluronic F127-RGD conjugate surface decoration	Cell line: A549 Animal model: Charles Foster/Wistar rats	Intravenous (Tail vein)	Achieved controlled drug release up to 72 h; demonstrated significantly higher cytotoxicity IC50 = 0.281 μg/mL compared to marketed CDDP 6.856 μg/mL, improved systemic exposure (4.6-fold higher AUC_(0-t)_ 4-fold longer t_1/2_ than Ciszest-50); exhibited high blood compatibility and minimal pulmonary/systemic toxicity.	Yadav et al. (2023)
Bismuthene nanosheets (2D Bi nanosheets)			Cell line: A549 lung adenocarcinoma cells Animal model: A549 xenograft-bearing BALB/c nude mice	Intravenous (tail vein) injection followed by 850 nm NIR irradiation and alternating magnetic field (50 Hz, 600 mA)	Exhibited soft magnetic and excellent photothermal properties, inducing complete in vitro cancer cell death through synergistic PTT and MH. Activated immunogenic cell death, promoted ferroptosis, significantly inhibited tumor growth, enhanced dendritic cell and NK cell infiltration, increased tumor temperature to ~47 °C, and showed high biocompatibility with negligible hemolysis and systemic toxicity.	Yilmazer et al. (2023)
Lipid nanoplatform incorporating NaLuF₄:Yb/Er@NaLuF₄ upconversion nanoparticles (UCNPs) and IR-1048 dye (UCILA)	IR-1048 (NIR-II photothermal agent) + CAR-NK-92MI immunotherapy	AS1411 aptamer (nucleolin-targeting), DSPE-PEGylated lipid coating	Cell line: A549 human NSCLC cells, L929 fibroblasts. Animal model: A549 tumor-bearing nude mice	Intravenous (tail vein) injection	The nanoplatform enabled penta-modal imaging with real-time temperature monitoring. NIR-II photothermal therapy significantly ablated tumors, while sequential CAR-NK immunotherapy eliminated residual tumor cells, resulting in complete tumor eradication, reduced tumor vasculature, prolonged survival, minimal systemic toxicity, and efficient biodistribution/metabolism.	Xu et al. (2021)
Hollow polydopamine/gold nanoparticle nanocomposite	DOX	–	Cell line: A549 human NSCLC cells. Animal model: BALB/c nude mice with A549 lung metastasis model (tail vein injection) and A549 subcutaneous xenograft model	Intratracheal administration	HPDA/Au@DOX (~164 nm) exhibited high drug loading (36.31%), encapsulation efficiency (90.78%), and excellent photothermal conversion (49.3%) with NIR-responsive drug release. NIR irradiation enhanced cellular uptake and cytotoxicity in A549 cells. Combined photothermal therapy and chemotherapy effectively inhibited cell proliferation, induced apoptosis, suppressed lung metastasis, and reduced tumor growth more than free DOX or HPDA/Au@DOX alone. Intratracheal administration enabled preferential lung accumulation with excellent hemocompatibility, minimal systemic toxicity, and no significant liver, kidney, or hematological toxicity.	Zhang et al. (2024)

## 5 Advances in lung cancer diagnosis

Accurate diagnosis of lung tumors is crucial because, in the early stage, lung cancer shares several symptoms with other respiratory conditions, such as bronchitis, bronchiolitis, and tuberculosis (Bhatt et al., 2012). Due to the similarity in their symptoms, it is often misdiagnosed with these diseases. Misdiagnosis can result in untreated lung cancer and may lead to serious complications. Imaging techniques play a major role in the detection, diagnosis, and management of lung tumors. Chest X-ray, computed tomography (CT), magnetic resonance imaging (MRI), and positron emission tomography (PET) are some imaging techniques that are used for the detection of lung tumors (Purandare and Rangarajan, 2015).

Among these techniques, Chest X-ray stands as the first-line investigation technique for detecting the preliminary stages of lung cancer due to its cost-effectiveness, accessibility, and minimal radiation exposure for the detection of cancer. However, its detection rate is typically below 20% (Kim and Kim, 2020). For patients at high risk or when lung cancer is not detected on chest X-rays, additional imaging techniques are utilized for further evaluation.

### 5.1 Conventional imaging and radiography

The most popular non-invasive method for lung cancer screening and staging is chest CT, which is a basic imaging technique for the assessment of lung tumor. Determining the dimensions, location, and characteristics of lung lesions which are encircled by lung tissue that is filled with air is very helpful (Beigelman-Aubry et al., 2016). Chest CT can also be used to evaluate metastases in the liver, adrenal glands, bone, and other areas of the thoracic cavity. In Korea, low-dose chest CT (LDCT) has become the standard for screening for lung tumor in high-risk adults since September 2019 (Arnon-Sheleg et al., 2020). Using lung doppler CT (LDCT) screening in place of traditional chest radiography improves the prognosis for patients with lung cancer by effectively identifying tiny lung nodules and facilitating early management. In comparison to chest X-rays, the U.S. National Lung tumor Screening Trial showed that LDCT screening reduces lung tumor-associated mortality in high-risk individuals by 20%. Individuals who have favorable results from LDCT should be assessed further using additional diagnostic techniques (Amicizia et al., 2023).

### 5.2 Computed tomography (CT) scan

To diagnose and treat lung cancer, computed tomography, sometimes referred to as computed axial tomography, is a highly effective method. X-rays and computer processing are used in this method to create intricate cross-sectional pictures of the body. Its primary objectives encompass enhancing diagnostic precision, enabling early detection, and streamlining radiological evaluation processes (Al-Naser and Tafti, 2024). CT excels in detecting pulmonary nodules, leveraging its high contrast resolution to identify subtle abnormalities indicative of early-stage cancer. However, it's noteworthy that many malignant nodules exhibit low contrast. This phenomenon is attributed to the diverse filters utilized to optimize image quality (Rubin, 2015).

### 5.3 Positron emission tomography-CT imaging (PET-CT)

PET-CT is an imaging method utilized primarily in oncology for the detection and localization of malignant cells within the body (Griffeth, 2005). This technique relies on the administration of positron-emitting radioisotopes, notably fluorine-18, which are incorporated into biologically active molecules, upon decay, these radioisotopes emit positrons, which subsequently interact with electrons, resulting in the production of gamma rays (Alauddin, 2011). PET cameras are employed to detect and record the gamma rays emitted during this process. By combining PET and CT imaging, structural information provided by CT scans can be integrated with functional data obtained from PET scans, enhancing the accuracy and specificity of cancer detection. The synergistic use of PET and CT modalities allows for comprehensive visualization and characterization of cancerous lesions, aiding in diagnosis, staging, and treatment planning. Lee et al., reported as a gold standard, endobronchial ultrasound-guided transbronchial needle aspiration (EBUS-TBNA) can classify mediastinal lymph node (L/N) metastasis in Korea with up to 87% false positive rates for PET-CT. This rate is significantly higher in patients with squamous cell carcinoma or pneumoconiosis who are older than 65. That same study did find, however, that the false positive rate was not substantially impacted by either a history of past or present TB. This is in contrast to earlier findings that suggested PET-CT's utility declined in regions where tuberculosis incidence was high (Lee et al., 2018).

### 5.4 Whole-body bone scintigraphy

Bone metastases affect between 30% and 40% of people with NSCLC, which worsens their quality of life over the remainder of their lives and gives them a worse prognosis. When combined with other pertinent clinical symptoms including bone pain, increased alkaline phosphatase, and hypercalcemia, whole body bone scintigraphy (WBBS) can be helpful in the diagnosis of bone metastases. The National Comprehensive Cancer Network recommendations do not advise frequent use of WBBS because of its high false positive rate caused by trauma, inflammation, and degenerative changes to the skeletal system. Furthermore, because bone scans are not included in the HIRA indication as imaging techniques used to detect lung cancer bone metastases, the use of WBBS for lung tumor is declining in Korea (Park et al., 2020; Yeo et al., 2018).

### 5.5 Fiberoptic bronchoscopy

Fiberoptic bronchoscopy (FBS) provides direct visualization of the bronchial anatomy, allowing the airway pathway to the target lesion to be established and the optimal site for tissue sampling with forceps or bronchial brushes to be identified. Commercial FBS has a diameter of around 6 mm, making it useful for detecting and biopsying lesions up to the subsegmental bronchus. FBS has a poor diagnostic yield, ranging from 20% to 60% (Moon et al., 2019). The lesion's size, location, and visibility are all significant characteristics that affect the diagnostic yield. Low yields are generally observed in peripheral lung regions when lung lesions with a diameter of less than 2 cm are present. It is suggested to perform three or more biopsies in order to obtain excellent diagnostic yields when dealing with endobronchial lesions located in central locations. The diagnostic yield for lesions that are not immediately evident by FBS can also be increased by transbronchial biopsy, bronchial cleaning, and bronchial brushing. A transbronchial lung biopsy guided by fluoroscopy can enhance the detection precision for peripheral lung lesions that are not accessible with standard FBS. Other fusion methods have also been developed and applied to increase detection yield, such as navigation bronchoscopy and EBUS.

### 5.6 Endobronchial ultrasound-guided transbronchial needle (EBUS-TBNA)

Originally developed to detect mediastinal lung metastases of lung cancer, EBUS-TBNA is currently often utilized for central lesions that are inaccessible to FBS, as well as for L/N evaluation. Usually, the EBUS-TBNA needle is introduced into the mass or lymph node through the wall of the trachea or major bronchus. Using real-time ultrasound imaging, the target lesion's location is verified during this process. With this low-complication rate technique, It is possible to assess the highest mediastinal, hilar, subcarinal, upper paratracheal, lower paratracheal, and interlobar L/Ns (Aziz, 2012; Kim et al., 2017). This approach does not, however, provide access to the para-aortic, aortopulmonary window, subaortic, paraesophageal, or pulmonary ligament L/Ns. EBUS-TBNA has a good diagnostic yield, with a 90% sensitivity and a 93% negative predictive value (Dong et al., 2013). EBUS-TBNA had a 77% sensitivity in confirming primary central lung cancer in individuals whose routine FBS test findings were negative (Tournoy et al., 2009).

### 5.7 Laboratory and molecular diagnostics

A major factor in the development, spread, and beginning of tumor is genomic change. The discovery of genetic changes that may influence the response to treatment in lung cancer has been made feasible by a number of technical developments in cancer genomic analysis platforms. As a result, research on genetics and genomics is being done extensively in the field of lung cancer, and variables including EGFR, ALK, and ROS1 have been found and are being exploited as targets for lung tumor treatment. Furthermore, information on gene mutations such as BRAF, VEGF, KRAS, RET, and MET is also utilized in cancer treatment, in addition to immunological markers such PD ligand 1 (PD-L1) and programmed cell death (PD)-1 (El-Telbany and Ma, 2012). Polymerase chain reaction-based tests: The groundbreaking technique known as polymerase chain reaction (PCR), developed in 1983, enables the in vitro amplification of substantial quantities of DNA with the use of two primers. PCR-based assays have undergone constant modification to facilitate the identification of driver mutations, like EGFR, in clinical settings (Garibyan and Avashia, 2013).

Next generation sequencing based tests: Next generation sequencing (NGS) is a process that divides a genome into several pieces, reads each fragment concurrently, and then combines the data acquired using bioinformatics methods to swiftly decode a substantial amount of genomic information (Park et al., 2018). Amplicon sequencing is utilized when in-depth reading and assay sensitivity assessment are needed, while hybrid capture sequencing is employed when evaluating whole genomes, full exomes, or sizable tailored panels. Numerous earlier studies have verified the use of targeted NGS panels. In the management of lung tumor, targeted NGS panels such as those for EGFR, KRAS, BRAF, and MET are often utilized (Garinet et al., 2018).

Real time-PCR: Real-time PCR using SYBR Green and real-time PCR utilizing a TaqMan probe are the two most used forms of real-time PCR techniques. Despite being the gold standard for identifying driver mutations, tissue biopsies can be invasive and difficult to acquire depending on the patient's state and the location or size of the tumor. A liquid biopsy from pleural effusion, bronchoalveolar lavage fluid, or plasma is usually less intrusive than a tissue biopsy. Liquid biopsies and real-time PCR have produced encouraging results in earlier research (Shin et al., 2017).

### 5.8 Artificial intelligence-enabled lung cancer diagnosis

The present clinical practice of early screening using chest CT scans is a laborious, subjective, and subject to inter-observer variability approach. The medical profession has come to recognize the substantial clinical and scientific utility of AI in assisting with pathological diagnosis, driven by recent advancements in AI and digital pathology. Multi-parametric clustering analysis, made possible by the usage of AI recognition technology, helps doctors screen for early-stage lung tumor while decreasing mistakes and improving problem-solving effectiveness. AI has advanced the identification, diagnosis, and treatment of lung cancer (Hosny and Aerts, 2019; M. Liu et al., 2023b). AI can help in the diagnosis of cytopathological (Kichenadasse et al., 2020), histopathological (Yu et al., 2016), lung cancer biomarker (Wang et al., 2019a) and prognosis in luna cancer (Wang et al., 2019b). At the moment, the FDA has authorized many AI programs for interpreting chest CT scans and CXRs, allowing AI systems to participate in the identification of lung cancer. After AI applications in radiology proved successful, the technology was extended to digitalized whole slide imaging (WSI) annotation. By identifying EGFR mutations and PD-L1 expression and integrating with additional data, such as clinical and demographic information, AI systems may be able to assist in decision-making. By anticipating medication response, the rate of tumor recurrence following surgery, radiation response, and side effects, AI systems also assist physicians in estimating the prognosis of their patients. For the near future, integrating AI technologies into the healthcare process is essential, despite certain remaining challenges (Chiu et al., 2022).

## 6 Theranostic nanomedicine: integrating diagnosis and therapy

Theranostic application represents a precision-medicine approach that integrates diagnostic imaging and targeted therapy into single platform. It refers to agents or systems that can both detect disease and deliver treatment, often using the same molecular target, ligand or nanocarrier. This enables real-time monitoring of drug delivery, tumor response, and treatment adjustment, addressing key challenges in lung cancer (Yang et al., 2026). The concept of administering medicines and contrast agents simultaneously at the site of action is thought to contribute to the individualization of modern medicine. Among the nano-carriers advancing theranostic therapy toward clinical use are liposomes, polymeric nanoparticles, polymeric micelle forms, polymers, lipids, proteins, carbon, nucleic acid, and metals are the building blocks of other nano-carriers (Choi and Kim, 2023).

A multifunctional theranostic H-dot nanoplatform was developed for image-guided lung cancer and lymph node metastasis (LNM) surgery combined with targeted combination therapy by Yin et al. (Yin et al., 2022). Gefitinib (Gef) and genistein (Gen) were successfully encapsulated into H-dots, while maintaining excellent water solubility and stable inclusion complex formation. The nanoplatform exhibited pH-responsive drug release, with enhanced Gef release under acidic tumor conditions. In vitro studies demonstrated that Gef/H-dot and Gen/H-dot significantly improved cytotoxicity against Lewis lung carcinoma (LLC) cells compared with free drugs, while the combined formulation showed strong synergistic antitumor activity and reduced the EC50 of gefitinib. In vivo biodistribution and pharmacokinetic studies revealed efficient tumor accumulation, minimal nonspecific uptake, rapid renal elimination, and favorable pharmacokinetic profiles. Dual-channel NIR fluorescence enabled precise intraoperative visualization of primary tumors and metastatic lymph nodes, facilitating image-guided surgical resection and pathological identification. In murine lung cancer models, combined Gef/H-dot and Gen/H-dot therapy markedly inhibited tumor growth, enhanced apoptosis, suppressed angiogenesis, proliferation, and inflammation through downregulation of VEGF, CD31, COX-2, and Ki-67, while increasing TUNEL-positive cells. Histopathological and biochemical analyses further confirmed excellent biocompatibility with reduced pulmonary and hepatic toxicity compared with free drug treatment (Fig. 8).

**Fig. 8:**
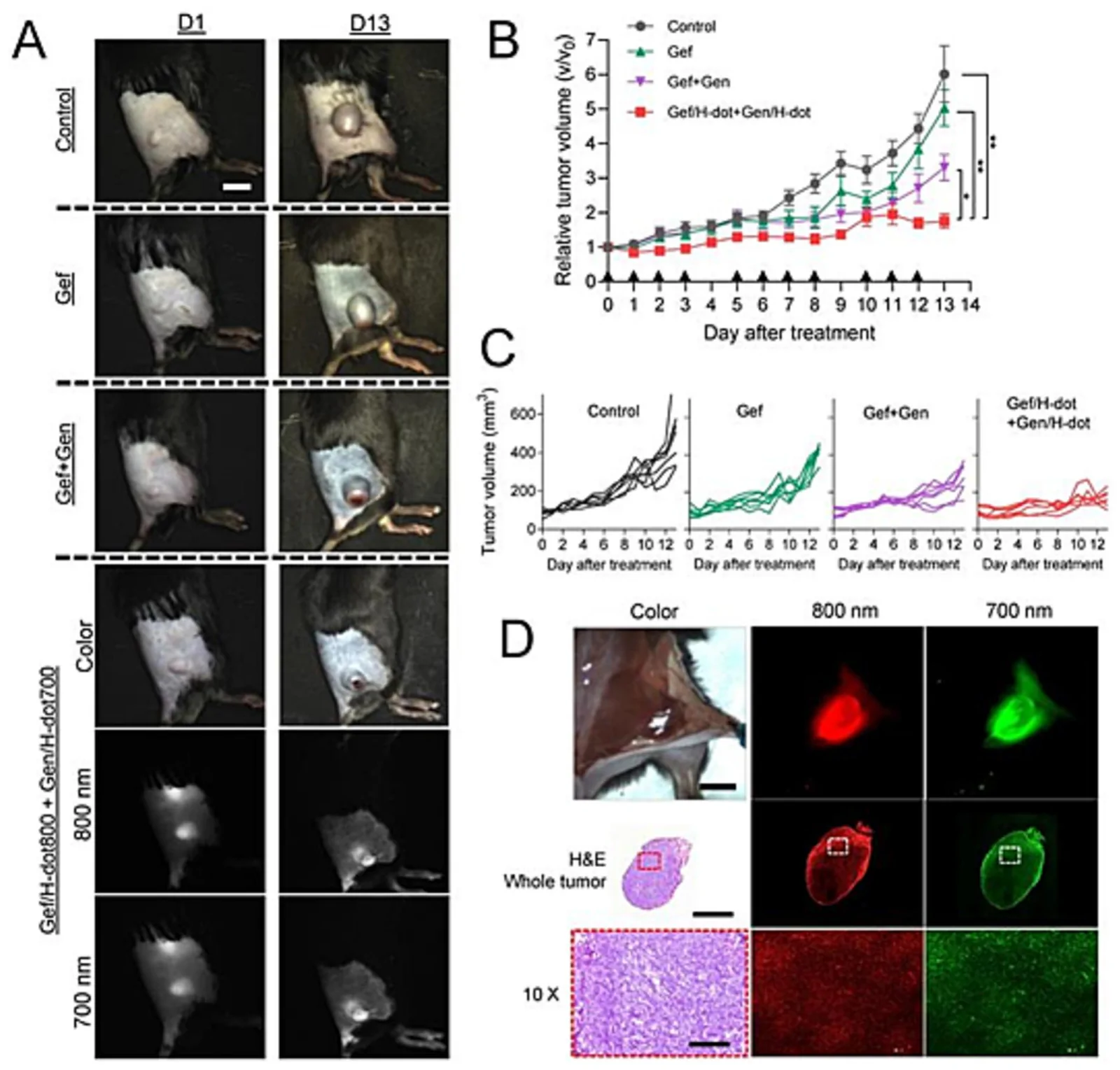
In vivo therapeutic efficacy of combination treatment with theranostic Gef/H-dot and Gen/H-dot compared with saline, Gef, and Gef + Gen in tumor-bearing mice (D13; *n* = 7/group). Black arrowheads indicate treatment days. (A) Representative color and NIR fluorescence images on days 1 and 13. (B) Tumor growth curves. (C) Individual tumor volumes at the end of treatment. (D) Intraoperative NIR fluorescence and microscopic images of resected tumors following combined Gef/H-dot800 and Gen/H-dot700 treatment. Scale bars: 5 mm (intraoperative), 2 mm (whole tumor), and 200 μm (10× microscopy). *p < 0.05, **p < 0.01. Adapted with permission from (Yin et al., 2022).

Muradova et al. fabricated bismuth-doped AGuIX nanoparticles were functionalized with cyclic Arg-Gly-Asp (cRGD) peptides, generating targeted AGuIX-Bi-cRGD nanocrystals that selectively bind integrin-overexpressing lung cancer cells (Muradova et al., 2025). The nanocrystals (~8.1 nm) retained excellent MRI contrast while exhibiting enhanced integrin-mediated cellular uptake and significantly increased radiation-induced DNA double-strand breaks compared with non-targeted AGuIX-Bi. Although both formulations showed minimal toxicity without irradiation, AGuIX-Bi-cRGD markedly improved radiosensitization in vitro. In LLC tumor-bearing mice, AGuIX-Bi-cRGD combined with fractionated radiotherapy (3 × 6 Gy) significantly suppressed tumor growth, induced complete tumor regression, prolonged survival, and showed greater efficacy than non-targeted nanoparticles. The targeted nanocrystals also demonstrated enhanced tumor retention, rapid renal clearance, and excellent biocompatibility. Additionally, treatment promoted immunogenic cell death by increasing HMGB1 expression and CD3^+^/CD8^+^ T-cell infiltration, highlighting the potential of AGuIX-Bi-cRGD as a targeted theranostic nanocrystal for MRI-guided radiotherapy and immunomodulation in lung cancer.

To enhance the radiation dose amplification (RDA) at the same time maintaining the positive MRI contrast of conventional AGuIX® nanoparticles, the high atomic number of bismuth (Bi^3+^) was introduced, resulting in second-generation AGuIX-Bi nanoparticles (Brown et al., 2023). Bi^3+^ significantly enhances the photoelectric effect causing more secondary electrons, deeper energy deposition and higher damage to DNA in radiotherapy. The optimized 30Gd:70Bi formulation had a hydrodynamic diameter of ~4.8 nm, which is ideal for renal clearance, and showed extended blood circulation and tumor targeting. In vitro, DNA double-strand breaks, apoptosis, and radiosensitization were significantly increased in NSCLC cells while maintaining similar levels of nanoparticle toxicity. AGuIX-Bi exhibited durable tumor accumulation both in vivo, with a negligible systemic toxicity and enhanced therapeutic effects, that included tumor growth delay and 33% complete regression upon radiotherapy. Farshbaf et al. combined Fe_3_O_4_ magnetic nanoparticles (MNPs), mesoporous silica nanoparticles (MSNs), and PDMAEMA/PNIPAAm polymers to create a multifunctional pH- and thermo-responsive magnetic nanocomposite (M-MNC) for targeted methotrexate (MTX) delivery and MRI-guided theranostics (Farshbaf et al., 2018). FT-IR showed successful functionalization of the nanoparticles and VSM analysis showed that the superparamagnetic property was retained with a saturation magnetization of 62.92 emu/g. The nanocomposites exhibited hydrodynamic diameters of 297–319 nm, pH-responsive surface charge reversal from negative (pH 7.4) to positive (pH 5), and improved particle dispersion. MTX loading achieved an encapsulation efficiency of 68% and drug loading of 13.6%. Drug release was minimal under physiological conditions (38.8% at pH 7.4, 37 °C) but markedly increased under acidic and hyperthermic conditions, reaching 92.3% at pH 5 and 40 °C, thereby demonstrating dual-stimuli-responsive release. In A549 cells, MTX-loaded M-MNCs showed enhanced cytotoxicity compared with free MTX, whereas blank nanocomposites exhibited minimal toxicity. Additionally, the nanocomposites produced concentration-dependent T_2_-weighted MRI contrast with an r_2_ relaxivity of 0.15 mM^−1^ ms^−1^, confirming their potential as a multifunctional theranostic platform for targeted cancer therapy. In another study, a facile one-step UV-assisted method was developed to synthesize a multifunctional graphene quantum dot-coated gold nanosphere cluster (Au@GQD-FA) for targeted cancer theranostics by Xuan et al. (Xuan et al., 2019). GQDs served as the reducing agent, stabilizer, and drug carrier, eliminating the need for toxic reagents. The ~50 nm Au@GQD nanoparticles were highly NIR absorbing and had high photothermal conversion efficiency (51.3%), excellent stability and efficient folic-acid conjugation for targeted active tumor targeting. DOX was successfully loaded with 94.4% EE and revealed that DOX exhibited dual-responsive release, releasing 44.6% at pH 5.4 and 80.8% under NIR irradiation, which provide precise chemo-photothermal therapeutic ability. The nanoplatform also exhibited an excellent photoacoustic (PA) and CT dual-modal imaging function. In vitro, the uptake in folate receptor positive HeLa cell was significantly enhanced and the hemocompatibility was excellent, with only minimal toxicity toward normal cells, both in the imaging and the release of the intracellular drug delivery in vitro assays. The probe was found to be well accumulated at the tumor site in vivo, to allow high contrast PA/CT imaging, and to show superior tumor ablation efficiency under NIR irradiation with synergistic chemotherapy and photothermal therapy, and markedly lower tumor recurrence than the monotherapies in vivo. The study of biosafety was performed by biodistribution, hematological, biochemical, and histopathological analysis, which revealed that Au@GQD-FA does not cause any significant damage to major organs, demonstrating the potential of Au@GQD-FA as a safe and effective multifunctional theranostic platform for targeted cancer diagnosis and therapy.

Theranostic nanomedicine is overall a promising approach to the management of lung cancer, which could combine targeted diagnosis, image-guided therapy and real-time monitoring of treatment in a single multifunctional platform. The addition of imaging modalities like MRI, NIR fluorescence, photoacoustic imaging and CT further enables accurate tumor localization, guidance for therapy and monitoring of therapeutic response. In total, these nanoplatforms demonstrate a platform approach that can facilitate the future of personalized lung cancer treatment by enabling accurate diagnosis and targeted treatment approaches. In spite of promising preclinical results, there is a need for additional optimization of long-term safety, large-scale production and clinical translation for these integrated nanotheranostic systems to be routinely used in clinical practice.

1. Relationships between Nanocarrier Design and Therapeutic Performance

The studies indicate that therapeutic performance is governed by the interplay between nanocarrier composition, surface characteristics, drug-release kinetics, and experimental conditions. Accordingly, enhanced cellular uptake or tumor inhibition cannot be attributed solely to particle size. Comparisons between independently developed formulations are influenced by differences in therapeutic payload, dose, cancer model, exposure duration, and administration conditions. The available evidence therefore supports formulation-specific associations rather than a universally optimal particle size or a consistent hierarchy of nanocarrier classes.

The relationship between particle size and biological performance is particularly evident when considered alongside carrier composition and surface properties. Curcumin-loaded solid lipid nanoparticles measuring 114.9 ± 1.36 nm enhanced uptake and cytotoxicity in A549 cells, although changes in drug stability and release prevented attribution of these benefits to size alone (Rahman et al., 2023). Similarly, GL67 lipoplexes measuring 311 ± 2 nm achieved approximately 67% siRNA uptake, whereas a smaller formulation measuring 144 ± 18 nm showed negligible uptake; however, lipid composition, surface charge, and complexation also differed (Jarallah et al., 2023). Gefitinib-loaded lipid carriers below 300 nm exhibited lower cytotoxicity than free gefitinib at 24 h but comparable activity at 48–72 h, highlighting the influence of release kinetics and exposure duration (Sherif et al., 2023). Collectively, these findings support evaluating particle size together with carrier composition, surface properties, and drug-release behaviour to guide formulation optimization and improve therapeutic performance.

The influence of carrier composition is particularly evident in drug-release behaviour. Sherif et al. demonstrated that modification of the liquid-lipid component of gefitinib-loaded nanostructured lipid carriers altered their physicochemical characteristics and release kinetics. The long-chain triglyceride formulation exhibited pronounced initial release, whereas the long-chain fatty acid formulation provided more controlled release. These findings indicate that lipid selection affects drug availability as well as nanoparticle properties. Sustained release should therefore be evaluated in relation to exposure duration and biological response rather than regarded as an intrinsically superior formulation characteristic (Sherif et al., 2023). *Co*-encapsulation of ETO and a cisplatin prodrug in poly(2-oxazoline) micelles improved drug retention, pharmacokinetics, and tumor delivery, accompanied by enhanced therapeutic outcomes (Wan et al., 2018). Together, these findings suggest that the significance of release control is best established through its relationship with therapeutic exposure and efficacy.

Surface engineering and combination delivery also influence therapeutic performance, although appropriate experimental comparisons are necessary to distinguish their respective contributions. Folate-functionalized nanostructured lipid carriers were designed for targeted, co-delivery of docetaxel and curcumin (Rawal et al., 2021). Similarly, the superior outcomes of *co*-loaded polymeric micelles relative to single-drug formulations or separately formulated agents support a contribution from co-formulation (Wan et al., 2018). Enhanced efficacy may therefore arise from interacting design features and should not be attributed exclusively to ligand functionalization or nanoparticle uptake. Arslan et al. combined CD56-directed targeting with co-delivery of irinotecan and the STAT3 inhibitor Stattic in PLGA-PEG nanoparticles, reporting enhanced cellular uptake and antitumor activity alongside preferential distribution to tumor tissue and lungs. This approach links receptor-directed delivery with modulation of a therapeutic signaling pathway (Arslan et al., 2023). Chen et al. integrated biotin functionalization, curcumin–erlotinib co-delivery, and photothermal activity within a MoS₂ nanoplatform. Comparison with the non-biotin-functionalized formulation supported enhanced tumor accumulation, whereas irradiation-dependent efficacy demonstrated an additional contribution from photothermal treatment (Chen et al., 2023). Collectively, these studies indicate that therapeutic benefit can arise from interacting targeting, pharmacological, and physical mechanisms; increased efficacy should therefore not be attributed exclusively to ligand functionalization or nanoparticle uptake.

The administration strategy further determines the formulation characteristics relevant to therapeutic assessment. Afatinib-loaded PLGA nanoparticles were formulated as a dry powder for pulmonary delivery, making aerosol performance an essential evaluation parameter (Vanza et al., 2023). Nebulized curcumin-loaded liposomes were assessed for photodynamic activity in cell-based and chick chorioallantoic membrane tumor models (Bender et al., 2024). These studies demonstrate the importance of compatibility between a formulation and its intended delivery method. Nevertheless, aerosol characterization or therapeutic activity in a chorioallantoic membrane tumor model does not independently establish drug deposition, distribution, or efficacy in human lung tumors. Zhang et al. provided complementary animal evidence using intratracheally administered DOX-loaded polydopamine/Au hollow nanospheres, which showed preferential pulmonary accumulation and enhanced therapeutic activity when combined with photothermal treatment. However, intratracheal administration should not be considered equivalent to aerosol inhalation, as the delivery procedures differ (Zhang et al., 2024). In a distinct approach, He et al. combined pulmonary-affinity PTX micelles with ambroxol to modify the pulmonary microenvironment, enhance lung accumulation, and increase therapeutic activity. The latter findings indicate that pulmonary exposure may also be influenced by carrier composition and adjunct treatment rather than by administration route alone (He et al., 2020). These studies support evaluating delivery performance through route-appropriate characterization, biodistribution, and biological outcomes, while avoiding direct efficacy rankings across dissimilar administration methods and models.

In theranostic systems, material composition can influence therapeutic activity independently of its function as a drug carrier. Brown et al. demonstrated that increasing the bismuth content of AGuIX-based nanoparticles enhanced radiation-associated DNA damage and antitumor efficacy while preserving magnetic resonance contrast (Brown et al., 2023). Such controlled comparisons provide stronger evidence of a composition-performance relationship than comparisons between unrelated nanoplatforms. Muradova et al. extended this approach through cRGD functionalization of AGuIX-Bi nanoparticles, reporting enhanced cellular uptake and radiosensitization compared with non-targeted nanoparticles, together with antitumor immune responses following irradiation. Considered together, these studies illustrate distinct contributions from material composition and surface targeting, although differences between their experimental models prevent direct comparison of efficacy (Muradova et al., 2025). Yin et al. demonstrated another dimension of theranostic utility through gefitinib- and genistein-loaded H-dots, which enabled fluorescence-guided identification and resection of lung tumors and metastatic lymph nodes alongside combination drug delivery (Yin et al., 2022). This provides a functional connection between imaging and therapeutic intervention, rather than demonstrating imaging capability alone. Accordingly, theranostic performance should be assessed by distinguishing imaging quality, delivery characteristics, and therapeutic outcomes, and by establishing whether imaging contributes to treatment localization, procedural guidance, or response assessment. However, preservation of imaging capability and enhancement of therapeutic efficacy represent distinct outcomes. The additional value of a theranostic platform should therefore also be evaluated according to whether imaging informs treatment delivery or response assessment.

Collectively, these studies support an integrated assessment of formulation characteristics, delivery performance, therapeutic exposure, and biological response. Enhanced uptake, increased cytotoxicity, tumor accumulation, and reversal of resistance should be distinguished as separate outcomes. Findings obtained in conventional cancer models do not independently demonstrate reversal of established drug resistance. Substantial differences in experimental controls and conditions further constrain direct comparison and generalization across studies. Future investigations should prioritize matched controls and clearly defined biological endpoints to identify the design features that reproducibly contribute to therapeutic benefit.

2. Lung Cancer Patents

The patents related to lung cancer diagnostics and therapeutics are summarized in Table 2. It contains several innovations for detecting biomarkers, immunotherapies, and treatment options. From new biomarkers and peptide treatments to advanced diagnostics and tailored therapies, patents cover various techniques. Every entry provides information about the patent title, claims, number, filing date, applicant and reference for further reading.

**Table 2:** List of lung cancer patents.

Patent Title	Claims	Patent No.	Patent Filing Date	Applicant	Ref
Novel liposomes with ginsenoside derivative as membrane material and preparations thereof	Liposomal chemotherapy. Ginsenoside derivatives form the membrane; PTX-containing formulations are evaluated in A549 lung cancer and drug-resistant A549/T models.	WO2019105408A1	Filed: 2018-11-29 Pub.: 2019-06-06	Xiamen Ginposome Pharmaceutical Co., Ltd.	D. Wang et al. (2019)
Dual-loaded liposomal pharmaceutical formulations	Dual-drug liposomes. Docetaxel in the lipid bilayer and DOX in the aqueous compartment enable co-delivery. NSCLC models are included in the experimental disclosure.	US10736845B2	Filed: 2016-03-03 Pub.: 2020-08-11	Cureport, Inc.	Zhu and Chen (2020)
Therapeutic constructs for co-delivery of mitotic kinase inhibitor and immune checkpoint inhibitor	Nanoparticle chemo-immunotherapy. Mesoporous silica constructs co-deliver a PLK1 inhibitor/siRNA and a checkpoint inhibitor, including anti-PD-L1. Lung cancer cell and mouse-model experiments support relevance.	WO2021011501A1	Filed: 2020-07-13 Pub.: 2021-01-21	Oregon Health & Science University; PDX Pharmaceuticals, Inc.	Yantasee et al. (2021)
Formulation of antibody-based drugs for treating lung cancer by inhalation	Novel pulmonary DDS; nanoparticle status unestablished. Claims inhalable anticancer-antibody formulations stabilized with excipients. Pulmonary delivery is the formulation innovation; the antibody alone is not classified as a nanomedicine.	US20210030868A1	Filed: 2020-07-29 Pub.: 2021-02-04	Anovent Pharmaceutical (U.S.) LLC	Huang et al. (2021)
Composition formulated into inhalable dosage forms for treatment of lung tumors	Novel pulmonary gene DDS. Inhalable, stabilized particles contain a vector encoding GM-CSF and bifunctional shRNA against furin. Claims include primary NSCLC/SCLC. Disclosed particles span micron/submicron sizes; do not describe the entire formulation as nanoscale.	WO2022055817A1	Filed: 2021-09-03 Pub.: 2022-03-17	Gradalis, Inc.	Shanahan et al. (2022)
Composition for treating lung cancer, particularly of NSCLC	RNA cancer vaccine formulation. RNA encoding tumor antigens including NY-ESO-1 and MAGE-A3 is used for lung-cancer vaccination. Cationic-protein complexation, including protamine, is disclosed.	US20220096616A1	Filed: 2021-07-19 Pub.: 2022-03-31	CureVac SE	Barner et al. (2022)
Therapeutic RNA for lung cancer	RNA lipoplex vaccine. Lung-tumor-antigen RNAs use cationic lipid formulations, including DOTMA/DOTAP and helper lipid DOPE. Antigen-presenting-cell targeting supports vaccination; direct lung-tumor deposition is not implied.	US20240415946A1	Filed: 2022-10-10 Pub.: 2024-12-19	BioNTech SE; TRON gGmbH	Sahin et al. (2024)
Immunotherapeutic constructs and methods of their use	Multicomponent nanoparticle immunotherapy. Constructs combine mitotic-kinase inhibition, checkpoint targeting and immune stimulation, including CpG. Lewis lung carcinoma models are disclosed. This extends beyond the two-component construct above.	US20250195641A1	Filed: 2025-02-06 Pub.: 2025-06-19	Oregon Health & Science University; PDX Pharmaceuticals, Inc.	Yantasee et al. (2025)
Individualized neoantigen vaccines for the treatment of NSCLC	Personalized mRNA-LNP vaccine. Claims postoperative neoantigen vaccination plus pembrolizumab after neoadjuvant therapy and resection. The mRNA formulation includes ionizable lipid, neutral lipid, sterol and PEG lipid.	WO2026064370A1	Filed: 2025-09-17 Pub.: 2026-03-26	Merck Sharp & Dohme LLC; ModernaTx, Inc.	Brown et al. (2026)
Combination therapies with hafnium oxide nanoparticles and pd-l1 antibodies for use in the treatment of NSCLC	Nanoparticle-assisted radio-immunotherapy. Intratumoral hafnium oxide nanoparticles are combined with radiotherapy, chemotherapy and PD-L1 blockade for NSCLC.	WO2026074471A1	Filed: 2025-10-01 Pub.: 2026-04-09	Janssen Pharmaceutica NV	Szapary et al. (2026)

## 7 Lung cancer clinical trials

Table 3 summarizes ongoing and completed clinical trials focused on lung cancer. It encompasses a variety of study types, including trials on screening methods, therapeutic interventions, and palliative care strategies. The trials cover different lung cancer subtypes and stages and aim to evaluate new treatments, diagnostic techniques, and patient care approaches. Each entry provides the study title, condition, outcomes, status, start date, and clinical trials identifier.

**Table 3:** List of clinical trials involving lung cancer treatments.

Study Title	Condition	Result/Outcomes	Status	Study start date	Clinical Trials Identifier
NBTXR3 and Radiation Therapy for the Treatment of Inoperable Recurrent Non-small Cell Lung Cancer	Inoperable, locoregionally recurrent NSCLC previously treated with radiotherapy	Safety, recommended Phase II dose, feasibility of reirradiation and antitumor response following intratumoral/intranodal administration of hafnium oxide nanoparticles with radiotherapy.	Active, not recruiting	10 February 2021	NCT04505267
Carbon Nanoparticle-Loaded Iron in the Treatment of Advanced Solid Tumor	Advanced solid tumors, including lung cancer	Safety, tolerability, pharmacokinetics and preliminary efficacy of repeated intratumoral CNSI-Fe(II) injections.	Recruiting	12 November 2024	NCT07433283
Paclitaxel Micelles for Injection versus Paclitaxel Injection, Each Combined with Cisplatin, for First-line Therapy of Advanced NSCLC	Advanced NSCLC	Objective response rate was significantly higher with polymeric micellar PTX plus cisplatin than with solvent-based PTX plus cisplatin: 50% versus 26%; *p* < 0.0001.	Active, not recruiting. Results published.	May 2015	NCT02667743
Irinotecan Hydrochloride Liposome Injection (LY01610) For Small Cell Lung Cancer	Small Cell Lung Cancer	A multicentre, single-arm, dose-ranging study evaluated liposomal irinotecan in 66 patients across three dose groups.	Completed	September 2020	NCT04381910
Study of Irinotecan Liposome Injection (ONIVYDE®) in Patients With Small Cell Lung Cancer (RESILIENT)	Small Cell Lung Cancer	Liposomal irinotecan did not demonstrate an overall-survival advantage over topotecan. This provides useful evidence that a nanocarrier formulation does not necessarily improve survival.	Completed	April 2018	NCT03088813

## 8 Clinical translation of nanomedicine in lung cancer

Clinical translation of nanomedicine in lung cancer requires evidence that formulation advantages translate into meaningful improvements in efficacy and safety. Polymeric micellar PTX (pm-Pac) is a Cremophor EL-free formulation comprising approximately 20-nm mPEG–PDLLA micelles (Shi et al., 2021). Following encouraging preclinical and phase I findings, a multicenter, randomized, open-label phase III trial enrolled 454 patients with advanced NSCLC to compare pm-Pac plus cisplatin with solvent-based PTX (sb-Pac) plus cisplatin as first-line therapy. Among 448 treated patients, pm-Pac significantly improved the independently assessed objective response rate (50% versus 26%; P < 0.0001) and median progression-free survival (6.4 versus 5.3 months; HR 0.63; *P* = 0.0001), with response benefits observed in both squamous and non-squamous tumors. Median overall survival was numerically longer (18.0 versus 16.4 months), although the difference was not statistically significant and survival data remained immature. Despite higher PTX doses, pm-Pac was associated with fewer treatment-related serious adverse events (9% versus 18%; *P* = 0.0090), whereas overall serious adverse event rates did not differ significantly. No acute hypersensitivity reactions occurred in the absence of antiallergic premedication. These findings support the clinical translation of polymeric micellar PTX. Zhang et al. compared a multicenter, randomized, open-label trial evaluating liposomal PTX (Lipusu) plus cisplatin (LP) versus gemcitabine plus cisplatin (GP) as first-line treatment in 540 patients with locally advanced or metastatic lung squamous cell carcinoma (Zhang et al., 2022). Among 534 treated patients, per-protocol analysis showed no significant differences in objective response rate (41.8% versus 45.9%), median progression-free survival (5.2 versus 5.5 months), or median overall survival (14.6 versus 12.5 months). However, LP resulted in fewer adverse event-related treatment interruptions (10.9% versus 26.4%; *P* < 0.001) and terminations (14.3% versus 23.1%; *P* = 0.011), with significantly lower incidences of grade ≥ 3 anemia and thrombocytopenia. Serious adverse event rates were similar between groups. Exploratory cytokine analyses associated lower baseline IL-8 levels and treatment-associated reductions in IL-8 with better clinical outcomes. These findings support LP as an alternative chemotherapy regimen with a favorable toxicity profile, although the cytokine findings require validation and recruitment exclusively from China limits generalizability.

The randomized phase III IMpower130 trial evaluated atezolizumab, an anti-PD-L1 antibody, combined with carboplatin and nanoparticle albumin-bound PTX (nab-PTX) versus chemotherapy alone as first-line treatment for stage IV non-squamous NSCLC (West et al., 2019). Among 679 patients without EGFR or ALK genomic alterations, atezolizumab significantly improved median progression-free survival (7.0 versus 5.5 months; HR 0.64; *P* < 0.0001) and overall survival (18.6 versus 13.9 months; HR 0.79; *P* = 0.033). Objective response rates were also higher (49.2% versus 31.9%), with longer response duration (8.4 versus 6.1 months). Benefits were observed across PD-L1 expression subgroups. However, grade 3–4 adverse events (81% versus 71%) and treatment-related serious adverse events (24% versus 13%) were more frequent with the combination, although no new safety signals emerged. These findings support integrating nab-PTX-based chemotherapy with immune checkpoint inhibition, demonstrating a survival benefit with the addition of atezolizumab.

Albumin-bound PTX is an example of a nanomedicine evaluated in a large randomized clinical trial. Socinski et al. compared a randomized phase III trial involving 1052 patients with advanced NSCLC, comparing weekly nanoparticle albumin-bound PTX (nab-PTX; 100 mg/m^2^) plus carboplatin every three weeks with solvent-based PTX plus carboplatin (Socinski et al., 2012). The approximately 130-nm albumin-bound formulation significantly improved the independently assessed objective response rate (33% versus 25%; *P* = 0.005), particularly in patients with squamous histology (41% versus 24%; *P* < 0.001). Median progression-free survival (6.3 versus 5.8 months) and overall survival (12.1 versus 11.2 months) were numerically longer but did not differ significantly, meeting the reported noninferiority criteria. Despite higher PTX exposure and administration without premedication, nab-PTX reduced the incidence of grade ≥ 3 sensory neuropathy (3% versus 12%) and neutropenia (47% versus 58%), although anemia (27% versus 7%) and thrombocytopenia (18% versus 9%) were more frequent. Severe neuropathy improved to grade 1 more rapidly (38 versus 104 days), alongside better patient-reported symptoms.

The randomized, open-label phase III RESILIENT trial compared liposomal irinotecan (70 mg/m^2^ every two weeks) with topotecan in 461 patients with small-cell lung cancer progressing on or after first-line platinum-based chemotherapy (Spigel et al., 2024). Liposomal encapsulation was designed to prolong irinotecan circulation and sustain intratumoral exposure to its active metabolite, SN-38. Liposomal irinotecan doubled the independently assessed objective response rate (44.1% versus 21.6%; nominal *P* < 0.0001) but did not significantly improve median overall survival (7.9 versus 8.3 months; HR 1.11; *P* = 0.3094) or progression-free survival (4.0 versus 3.3 months). Response duration was similar (4.1 versus 4.2 months). Grade ≥ 3 treatment-related adverse events were less frequent (42.0% versus 83.4%), with lower hematological toxicity but greater gastrointestinal toxicity, particularly diarrhea. These findings demonstrate improved tumor response and reduced severe treatment-related toxicity. Collectively, these trials demonstrate that nanomedicine can improve tumor response and selected tolerability outcomes in lung cancer, although these advantages do not consistently translate into prolonged survival. Survival benefits observed with chemoimmunotherapy reflect the addition of immune checkpoint inhibition rather than an independent effect of the nanocarrier.

## 9 Future perspectives

Ongoing research on smart nanomedicine for lung cancer should establish whether improvements in formulation performance produce reproducible therapeutic benefits in clinically relevant settings. The studies reviewed demonstrate enhanced drug delivery, cellular uptake, and antitumor activity in lung cancer cell lines and animal models, including selected drug-resistant models. However, increased cytotoxicity or tumor accumulation alone does not establish reversal of therapeutic resistance. Investigations addressing resistance should demonstrate restored treatment sensitivity in models with defined resistance mechanisms, using appropriate free-drug, carrier, and combination-treatment controls. Similarly, studies of ligand-functionalized and stimuli-responsive systems should establish whether receptor recognition or stimulus-dependent release contributes to therapeutic efficacy beyond that achieved with otherwise comparable nanocarriers.

Translation requires evaluation across models that better represent the molecular diversity, stromal barriers, and immune environments of human lung cancer. Studies should examine how variable tumor vascular permeability, protein-corona formation, mononuclear phagocyte system clearance, and limited intratumoral penetration influence delivery and treatment response. Pharmacokinetics, biodegradation, immunogenicity, repeated-dose toxicity, and long-term material retention require systematic assessment. Manufacturing development should establish reproducible particle characteristics, drug loading, release behaviour, stability, and scalable production consistent with regulatory quality requirements. Ultimately, comparative clinical studies must determine whether these platforms improve survival, tolerability, or quality of life and identify the patient populations most likely to benefit.

## 10 Conclusion

The reviewed evidence demonstrates that nanocarrier formulations can improve drug solubilization, delivery, and antitumor activity in experimental lung cancer models. Selected clinical formulations have also improved tumor response or specific tolerability outcomes, although these benefits have not consistently translated into longer survival. However, our critical appraisal shows that most preclinical studies demonstrate improved delivery, uptake, and cytotoxicity in drug-sensitive models, whereas direct evidence for reversal of drug resistance is limited to a small number of studies in resistant models. Validating the resistance-reversal potential of these platforms is a key priority. With the advent of nanotechnology, biomaterials, and recent imaging techniques at the facility level, drug carriers have been developed that can deliver therapeutic agents directly to tumor regions. These drug delivery methods could help deliver more drugs to lung cancer cells. The battle against lung cancer is very promising, especially if combined with drug delivery technology and gene therapy, immunotherapy, and other innovative therapies. To effectively tackle the challenges posed by cancer heterogeneity and activate the full potential of the immune system, targeted medication delivery combined with advanced therapy might be the key. In the future, the role of individualized medicine in treating lung cancer will grow. Customized drug delivery devices tailored to specific cancer characteristics and genetic mutations can result in more personalized and effective treatments. Still, there are challenges to address, such as obtaining regulatory authorization, implementing research findings in medical facilities, and expanding manufacturing capacity. To surmount these challenges and accelerate the translation of new drug delivery methods into clinical practice, continued research and clinical work will require the efforts of all stakeholders involved. Nanocarrier-based approaches have demonstrated antitumor activity in experimental lung cancer models. However, as research and clinical trials continue, even more beneficial and personalized treatment options will be expected, which will lead to better health and quality of life. We believe that these technologies will be very useful in the fight against this aggressive form of lung cancer and possibly add to the therapeutic efficacy in the future when we hope to be able to cure this form of cancer.

## CRediT authorship contribution statement

**Viola Colaco:** Writing – original draft, Conceptualization. **Ashutosh Gupta:** Writing – original draft, Conceptualization. **Sandesh Ramchandra Jadhav:** Writing – original draft. **Aditya Dev Rajora:** Writing – original draft. **Moumita Saha:** Writing – original draft. **Sangeeta Adile:** Writing – original draft. **Amrita Arup Roy:** Writing – original draft. **Soji Soman:** Writing – original draft. **Sudheer Moorkoth:** Writing – review & editing. **Deepanjan Datta:** Writing – review & editing. **Srinivas Mutalik:** Writing – review & editing. **Bhupendra G. Prajapati:** Writing – review & editing. **Ritu Kudarha:** Writing – review & editing, Supervision. **Namdev Dhas:** Writing – review & editing, Supervision, Software, Conceptualization.

## Declaration of competing interest

The authors declare that they have no known competing financial interests or personal relationships that could have appeared to influence the work reported in this paper.

## Data Availability

No data was used for the research described in the article.
